# Gender Disparity in Expression of Sarcopenia in Haemodialysis Recipients: Analysis from the FITNESS Cohort

**DOI:** 10.1155/2023/5885059

**Published:** 2023-06-17

**Authors:** Benjamin M. Anderson, Daisy V. Wilson, Muhammad Qasim, Gonzalo Correa, Felicity Evison, Suzy Gallier, Charles J. Ferro, Thomas A. Jackson, Adnan Sharif

**Affiliations:** ^1^Department of Nephrology and Transplantation, Queen Elizabeth Hospital, Birmingham, UK; ^2^Institute of Inflammation and Ageing, University of Birmingham, Birmingham, UK; ^3^Department of Healthcare for Older People, Queen Elizabeth Hospital, Birmingham, UK; ^4^Institute of Immunology and Immunotherapy, University of Birmingham, Birmingham, UK; ^5^Hospital Del Salvador, Santiago, Chile; ^6^Department of Health Informatics, Queen Elizabeth Hospital, Birmingham, UK; ^7^PIONEER HDR-UK Hub in Acute Care, Edgbaston, Birmingham, UK; ^8^Institute of Cardiovascular Sciences, University of Birmingham, Birmingham, UK

## Abstract

**Background:**

There has been little exploration of the interplay between sarcopenia and frailty in haemodialysis, particularly regarding gender difference. We aimed to (1) assess whether ultrasound-derived low muscle mass (LMM) and sarcopenia are more common in male or female haemodialysis recipients; (2) assess whether age influences any observed gender difference, and (3) explore the interplay between sarcopenia, frailty, and gender in haemodialysis recipients.

**Methods:**

This was an exploratory analysis of a subgroup of adult prevalent (≥3 months) haemodialysis with frailty phenotype (FP) scores. Bilateral anterior thigh thickness (BATT) was obtained according to an established ultrasound protocol. Associations with frailty were explored via both linear and logistic regressions for BATT, LMM, and sarcopenia with a priori covariables, stratified by gender.

**Results:**

In total of 223 studies, participants had ultrasound measurements. Males showed greater prevalence of LMM. On adjusted analyses, LMM was associated with lower hand grip strength in males (*β* = −4.17; 95% C.I. −7.57 to −0.77; *P*=0.02), but not females (*β* = −1.88; 95% C.I. −5.41 to 1.64; *P*=0.29). LMM was also associated with slower walking speed in both males (*β* = −0.115; 95% C.I. −0.258 to −0.013; *P*=0.03) and females (*β* = −0.152; 95% C.I. −0.300 to −0.005; *P*=0.04). Sarcopenia was associated with greater odds of frailty on adjusted models in males (OR = 9.86; 95% C.I. 1.8 to 54.0; *P*=0.01), but not females (OR = 5.16; 95% C.I. 0.22 to 124; *P*=0.31).

**Conclusions:**

The clinical expression and significance of sarcopenia differ substantially between males and females on haemodialysis. Further work is required to elucidate underlying mechanisms and guide tailored treatment.

## 1. Introduction

Frailty is a syndrome of increased vulnerability to poor resolution of homeostasis after stressor events [1], associated with negative outcomes including mortality, hospitalisation, and disability [2]. The gold standard frailty diagnostic tool is the comprehensive geriatric assessment (CGA) [3]. This is rarely used in research practice due to cost and logistical barriers; instead, tools such as the frailty phenotype (FP) are often used [4].

Sarcopenia is a progressive and generalised disorder of skeletal muscle, defined by low muscle mass (LMM) and low muscle strength, and can contribute towards frailty [5]. Gold standard measurements include dual energy X-ray absorptiometry (DEXA) or computed tomography (CT), but ultrasound has emerged as a validated alternative that is not affected by timing in relation to dialysis [6–10].

There is heterogeneity in reports of gender differences in prevalence of both LMM and sarcopenia. A greater proportion of females than males had DEXA-derived low muscle mass in non-CKD (chronic kidney disease) participants aged >50 years [11], but this difference was not observed when the sample included participants aged ≥20 years [12]. However, systematic reviews of studies in nursing home residents [13] and the general population [14] found sarcopenia rates were similar between genders.

Heterogeneity is also observed in haemodialysis recipients, with reports of DEXA data ranging from females having higher proportions of sarcopenia [15] to LMM and sarcopenia being more prevalent in males [16] and to no gender difference at all. [17] There is disagreement between studies as to whether BIA-derived LMM and sarcopenia is [6, 18] or is not [19–21] more common in males than females on haemodialysis, though studies observing no significant difference may have lacked power. Other studies have defined LMM/sarcopenia from within-cohort medians rather than from healthy young adults as per European Working group on Sarcopenia in Older People (EWGSOP) guidelines [5, 22, 23].

Therefore, questions remain unanswered about the gender-specific prevalence of LMM and sarcopenia in haemodialysis recipients. To address this uncertainty, the aims of this study were to (1) assess whether ultrasound-derived LMM and sarcopenia are more common in male or female haemodialysis recipients, (2) assess whether age may influence any observed gender difference, and (3) explore the interplay between sarcopenia, frailty, and gender.

## 2. Materials and Methods

The FITNESS study follows a cohort multiple randomised controlled trial (cmRCT) design [24], the full protocol for which has been described elsewhere [25]. The study protocol was subject to favourable opinion by the South Birmingham Research Ethics Committee (Ref: 17/WM/0381) and was conducted in accordance with the Declaration of Helsinki. This article describes analyses from the cohort study phase of FITNESS.

### 2.1. Study Setting

Patients were recruited from a single nephrology centre located in Birmingham, England, with a diverse range of ethnic and socioeconomic groups. Eligible patients were identified by interrogation of hospital electronic patient records (EPR) and from discussion with clinicians at each dialysis unit. Eligible patients were approached, given written and verbal information about the study, and given sufficient opportunity to consider the information before giving their consent to join the cohort study.

### 2.2. Eligibility Criteria

Inclusion criteria were adults aged 18 and over, receiving regular haemodialysis for at least 3 months' duration and the ability to give informed consent. The exclusion criteria included inpatient care within 4 weeks of recruitment unless for vascular access purposes to avoid confounding of baseline data with frailty secondary to recent hospitalisation. For this analysis, bilateral lower limb amputation was another exclusion criterion.

### 2.3. Baseline Assessment

Baseline assessments of all study participants took place before and during one of their usual dialysis sessions. To negate the potential effect of the long break from dialysis upon frailty measurements, we avoided the first haemodialysis session after the weekend interval. Here, participants were dialyzed twice weekly; the dialysis session after the shortest interval was chosen for baseline assessment.

Physical assessments took place immediately before connection to dialysis on the participants' routine dialysis session. Grip strength was assessed using hand grip dynamometer (Grip-D, Takei Scientific Instruments, Japan), with arm resting at the side of the patient with the elbow in extension and the wrist in the neutral resting position. A practice grip was taken with results discarded and then one summative grip on each hand, for which the participant was encouraged to give maximum effort. Both scores were noted, but the greater of the two scores was taken for subsequent analysis.

Walking speed was measured over 4 m from a standing start; usual walking aids were permitted. If the participant was not able to complete the 4 m distance, no walking speed was calculated, and a deficit was registered for this component of the relevant frailty scores.

Once dialysis started, study participants completed a number of assessments supplemented by interrogation of electronic patient records. Full details are outlined in our methodology paper [25].

### 2.4. Ultrasound Measurements

Following baseline frailty assessment, further verbal consent was sought for ultrasound assessment. Ultrasonographic measurement took place during the participants' regular dialysis session by or under the direct supervision of the first author. Patients were positioned sitting at an angle of ≤45° with knees resting comfortably upon a cushion near the natural 10° to 20° resting position. Participants were instructed to relax during the examination. Scanning followed a protocol established in previous work [10], with subcutaneous tissue, vastus intermedius, and rectus femoris depth all captured in a single transverse plane at anterior midthigh. This was defined as 50% of the measured distance between greater trochanter and lateral epicondyle of the femur. Images and depth measurements were obtained using a Phillips Lumify L12-4 transducer via the Phillips Lumify app (Koninklijke Philips, Netherlands) on its factory musculoskeletal settings, with a power of −0.3 dB and gain of 50. The bilateral anterior thigh thickness was calculated as the sum of bilateral rectus femoris and vastus intermedius anterior-posterior depth, or double the unilateral rectus femoris and vastus intermedius depth in instances of unilateral lower limb amputation, or of dialysis access (e.g., femoral line or arteriovenous graft) restricting adequate exposure of the area to be scanned.

Thresholds for LMM were 38.53 m and 54.36 mm for females and males, respectively, derived from previous work, representing two standard deviations below the mean of gender-specific healthy volunteers as per EQGSOP2 guidelines [5, 10]. Low grip strength was <27 kg for males and <16 kg for females; slow walking speed was set as <0.8 ms^−1^ [5]. Sarcopenia was defined as low muscle mass and low grip strength; severe sarcopenia was assigned when low muscle mass, low grip strength, and slow walking speed were all present. Frailty assessment is detailed in Supplementary Table 1. Participants were considered not frail by FP if their total score was 0, vulnerable if their total score was 1-2, and frail if their score was 3–5.

### 2.5. Recruitment

As this was an exploratory study on a subgroup of FITNESS participants, no power calculation was applied to this part of the study.

### 2.6. Statistical Analysis

Statistical analyses were performed using STATA 17 (StataCorp. 2019. Stata Statistical Software: Release 17. College Station, TX: StataCorp LLC). Correlations were calculated using Spearman's rank correlation coefficient. Agreement was assessed with Cohen's Kappa and was rated as >0.9, almost perfect agreement; 0.8–0.9, strong; 0.6–0.79, moderate; 0.4–0.59, weak; 0.21–0.39, minimal; and ≤0.2, no agreement. Differences between groups were assessed using unpaired *T*-tests on transformed-normal continuous data and Chi-squared or Fisher's exact for proportions as appropriate.

Associations with continuous outcomes were analysed using linear regression, having satisfied the linear assumption via visual comparison of observed versus Lowess fit lines on scatter plot and augmented component plus residual plots. Robust standard errors were specified to account for heteroscedasticity. Multicollinearity was excluded on all analyses by variance inflation factor <10. Odds ratios for frailty were obtained by logistic regression.

Linear and logistic regressions were performed both unadjusted and adjusted for a priori covariables chosen for proven or suspected association for the outcome of interest. For frailty, these were introduced in a stepwise manner; Model 1 included age, ethnicity, gender, education level, index of multiple deprivation (IMD) quintile [26, 27], self-reported social support (yes/no), and haemodialysis vintage. Model 2 added to these self-reported health today (Euroqol 5D Visual Analogue Scale, EQVAS) [28], self-reported health change, PHQ-9 score [29], cognitive impairment (Montreal cognitive assessment [MoCA]) [30], and Charlson comorbidity index (CKD omitted). Model 3 added self-reported slow walking speed (from GP Physical Activity Questionnaire) [31] and use of walking aids (yes/no). A priori covariables for muscle strength/function were BMI, ultrafiltration volume remaining at time of scan (ml/kg dry weight), ethnicity, age, IMD quintile, Charlson index, self-reported health change, physical activity index (PAI) derived from GP physical activity questionnaire [31, 32], use of walking aids (yes/no), EQVAS, and haemodialysis vintage (months).

Missing IMD quintiles were handled via a dummy variable. All other missing data were handled via listwise deletion, as <1% of these data were missing. A *P* value <0.05 was considered significant.

## 3. Results

**Figure 1:**
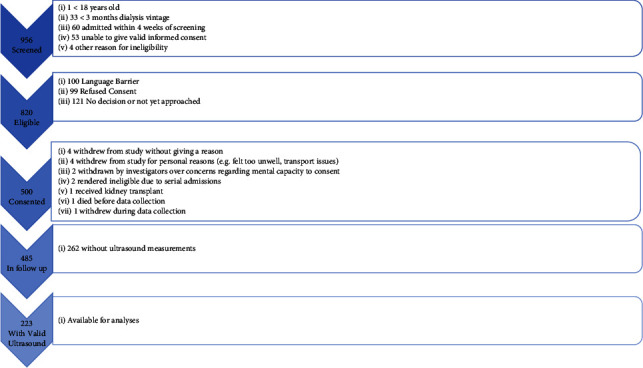
PRISMA flowchart of study participation.

Figure 1 shows a flowchart of recruitment to the FITNESS study. In total, 485 participants underwent frailty assessment and entered follow-up, of which 223 had valid ultrasound measurements. Of those with valid ultrasound measurements, the median FP was 2 (IQR 1–3) and 34% of participants were frail.  Table 1 shows key demographics stratified by gender.

### 3.1. Prevalence of Low Muscle Mass and Sarcopenia

Mean BATT was 49.4 mm (95% C.I. 47.0 to 51.8) in males and 44.6 mm (95% C.I. 41.7 to 47.6) in females (*P* for difference: 0.01).  Table 2 shows that LMM was significantly more prevalent in males. There were no statistically significant differences in muscle function between genders, but there was a nonsignificant trend towards more sarcopenia in males (36.2% versus 24.7%; *P*=0.07). BATT was associated with walking speed for males (Spearman's *ρ* = 0.362; *P* < 0.01) but not females (*ρ* = 0.126; *P*=0.28). BATT was associated with HGS in both males (*ρ* = 0.447; *P* < 0.01) and females (*ρ* = 0.295; *P* < 0.01).

Supplementary Table 2 shows higher prevalence of frailty, low grip strength, and slow walking speed in participants without ultrasound measurements versus those with ultrasound data. These differences were similarly maintained between genders.

### 3.2. Influence of Age on Gender Difference

**Figure 2:**
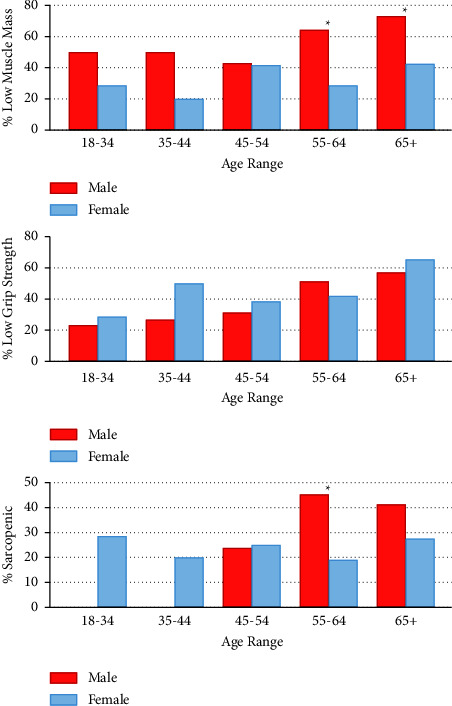
Prevalence of low muscle mass, low grip strength, and sarcopenia by age and gender. ^*∗*^ = significant at *p* < 0.05 level by Chi-squared or Fisher's exact, as appropriate.

Figure 2 shows that prevalence of low grip strength, LMM, and sarcopenia all remained relatively stable for females across age ranges, whereas for males, prevalence of each rose with age. When divided into age categories, males in the 54–65 and >65 age groups had significantly higher prevalence of LMM than females as shown in Table 3. As there were relatively few participants in each of the youngest three age categories, analyses were rerun by age tercile which demonstrated a similar pattern of LMM prevalence across genders, as shown in Table 4.

### 3.3. Association between Muscle Size and Function

Low muscle mass was associated with slower walking speed for males (*β* = −0.226; 95% C.I. −0.347 to −0.105; *P* < 0.01), but not females (*β* = −0.122; 95% C.I. −0.254 to 0.009; *P*=0.07) on simple linear regression. Low muscle mass was associated with poorer HGS on simple linear regression for both males (*β* = −7.64; 95% C.I. −10.7 to −4.62; *P* < 0.01) and females (*β* = −5.19; 95% C.I. −8.17 to −2.20; *P* < 0.01).

**Figure 3:**
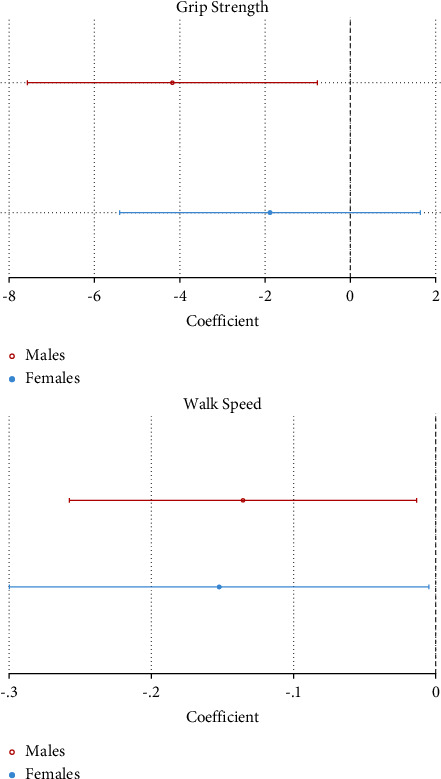
Multiple linear regression of grip strength (kg) and walking speed (ms^−1^) by low muscle mass.

Table 5 and Figure 3 show that walking speed was lower in both males and females with LMM on multiple linear regression, but only males with LMM showed lower HGS. Full multiple linear regression models, including those with BATT as an independent variable, are shown in Supplementary Tables 3–6.

### 3.4. Association between Muscle Size and Frailty

**Figure 4:**
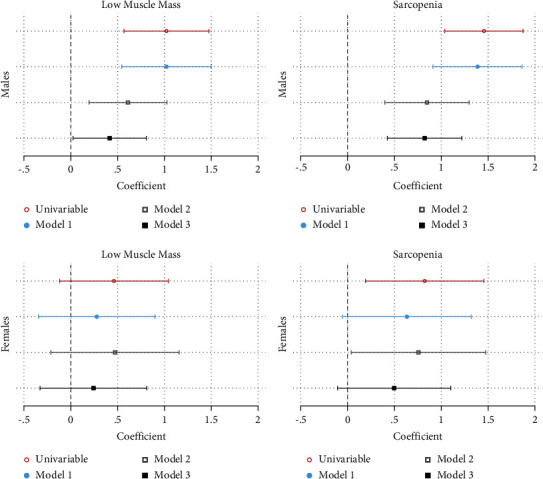
Simple and multiple linear regression models of frailty phenotype score by low muscle mass and sarcopenia (EWGSOP definition).

Figure 4 shows that in males, LMM and sarcopenia were associated with higher FP scores on all simple and multiple linear regression models. In females, LMM did not demonstrate significant association with FP scores on any model, whereas sarcopenia was associated with increased FP scores for simple linear regression and Model 2 only. Full model results, including models involving BATT, are shown in Supplementary Tables 7–12.

**Figure 5:**
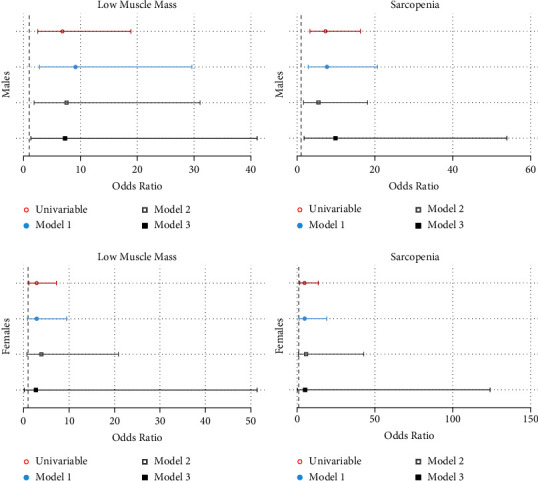
Univariable and multivariable logistic regression models of FP frailty by low muscle mass and sarcopenia (EWGSOP definition).

Figure 5 shows that on logistic regression in males, LMM and sarcopenia were associated with increased odds of frailty on all models. Females with LMM were more likely to be frail on unadjusted analyses but lost this association on all adjusted models. Sarcopenia in females was associated with increased odds of frailty on unadjusted analysis and Model 1 but lost this association in Models 2 and 3. Full models are shown in Supplementary Tables 13–18.

### 3.5. Constituents of Frailty Phenotype

Table 6 shows a slow walking speed was more common amongst females than in males. Furthermore, there was greater prevalence of low energy expenditure amongst females in the total FITNESS cohort and those without ultrasound measurements, but there was no difference in energy expenditure between males and females for participants with valid ultrasound measures.

## 4. Discussion

There are conflicting reports of gender-specific prevalence of sarcopenia and LMM in haemodialysis recipients, and the relationship between gender and age upon sarcopenia has yet to be fully explored. This is important as the greater prevalence of LMM and sarcopenia in males described in haemodialysis literature is incongruent with the general population, where there is greater prevalence of LMM in females in older age groups [11, 12]. In this study, we confirm that LMM is more common in male haemodialysis recipients and that there are also trends towards greater prevalence of sarcopenia in males. These gender differences appear driven by significant differences in older age groups; in younger haemodialysis recipients, no such differences between genders were observed. The links between muscle mass and function are stronger in males than females on haemodialysis. Furthermore, there are robust associations between frailty and LMM/sarcopenia in males, but not in females. These results suggest that the sarcopenic and frailty phenotypes differ between genders. Further work should explore any mechanistic differences in sarcopenia between genders and potential gender-specific mitigation for this important syndrome.

There is much interest in testosterone as a protective mechanism against sarcopenia and frailty. The majority of males receiving haemodialysis are testosterone-deficient, and testosterone levels fall with age [33–35]. Conversely, in a small observational study of haemodialysis recipients, all females enrolled had normal or supranormal testosterone levels [36]. Chiang et al. found low free testosterone in male; haemodialysis recipients were associated with low muscle mass, sarcopenia, and FP frailty both at baseline and at 12 months [37]. Our analyses demonstrate that gender differences in LMM among haemodialysis recipients appear to be driven largely by high prevalence in older men. In the context of current research into testosterone deficiency in haemodialysis, we may speculate that the effects of advanced CKD, uraemia, and haemodialysis itself may be synergistic with age to drive testosterone levels in older male haemodialysis recipients below protective levels. However, whilst testosterone replacement has been shown to increase muscle mass, HGS and physical function scores in men and women on haemodialysis, [38, 39] and quality of life in men, [40] no large scale studies have explored the relationship between frailty and testosterone deficiency in haemodialysis recipients. Furthermore, studies of testosterone replacement in older men in the general population have failed to demonstrate improved muscle performance despite improvements in strength and muscle mass [27, 41, 42]. We must therefore be cautious in ascribing gender differences to testosterone alone. Furthermore, there are legitimate concerns regarding the cardiovascular risk associated with testosterone supplementation in an already high-risk cohort [27].

Our work raises an interesting question: if testosterone deficiency is associated with sarcopenia and/or frailty in males on haemodialysis, then what of females? Female haemodialysis recipients exhibit higher prevalence of frailty compared to males, true even of the FP, the frailty score with the greatest musculoskeletal emphasis [43]. Males in our cohort exhibit higher prevalence of LMM and a trend towards greater sarcopenia. Furthermore, the link between sarcopenia and frailty appears more robust in males. We must take care not to conflate sarcopenia and frailty [5], but we must also question how a disparity in prevalence of LMM, sarcopenia, and FP frailty arises between genders. One explanation may be selection bias; FITNESS participants with ultrasound measures were less frail than those without, but this was true of both males and females and would not satisfactorily explain the discrepancy. Our data also show that female haemodialysis recipients are more likely to have slow walking speed and low energy expenditure than their male counterparts. This may explain why FP frailty is higher in females despite the lower prevalence of sarcopenia, but in turn raises questions about the mechanisms behind such differences. On this issue, there is remarkably little in the available literature to guide us. In the general population, resistance exercise but not moderate-to-high physical activity reduced incident sarcopenia for men, but the opposite was true for women [44]. An English older adult cohort found moderate and vigorous activity in males, but only vigorous activity in females was associated with reduced sarcopenia [45]. Therefore, there may be gender differences in sarcopenia expression and mitigation in nonrenal populations, which appears congruent with our findings. We suggest that further work is required to elucidate the differential mechanisms behind frailty and sarcopenia in males and females receiving haemodialysis, and why there appears to be such a different epidemiological pattern to sarcopenia in haemodialysis compared with other populations. A comprehensive systematic review was performed by March et al. who have shown that intradialytic exercise shows promise in mitigating for many components of the sarcopenia phenotype in all genders combined [46]. Clinicians may therefore feel this represents a pragmatic approach whilst detailed mechanistic study of gender differences is awaited to guide more tailored management.

Limitations of this study include that the whole FITNESS cohort did not complete ultrasound assessment. The ultrasound scanner was not procured until after the first 193 participants were recruited, and a further 69 refused or were unable to be scanned. Those not scanned were significantly frailer than those with ultrasound measurements. Scanning took place during haemodialysis sessions, in order to reduce the time commitment of each participant for the study. This may have resulted in the frailest being unable to participate in this aspect of the study due to concerns regarding mobility and adequate exposure for scanning, reducing the generalisability of our findings. In a further limitation, some of the 95% confidence intervals cross the point of no effect by small margins, most notably in the trend towards greater sarcopenia in males, raising the possibility of type II error. Participant numbers in younger adult age groups were also low. We attempted to mitigate for this by additional analyses using age terciles, but in so doing combined all participants <57-years into one group. It therefore remains challenging to draw conclusions on the nature of sarcopenia in young adult haemodialysis recipients on these data alone. Ultrasound is a relatively new method of determining low muscle mass, and there is a lack of standardisation [47]. As such, we recommend caution when comparing our results to other cohorts that may utilise differing methodologies. There are greater numbers of males within this analysis, though the gender proportions are comparable both to the broader FITNESS cohort, [43] and of renal replacement therapy recipients within Birmingham and the UK as a whole [48].

To conclude, low muscle mass is more common and there is a trend towards sarcopenia in male versus female haemodialysis recipients. However, frailty is more common in female haemodialysis recipients, driven by significantly greater prevalence of slow walking speed and low energy expenditure. Our work suggests there is a need to explore the mechanisms of gender difference underlying sarcopenia and frailty in haemodialysis recipients and to consider gender-specific management strategies.

## Figures and Tables

**Table 1:** Baseline characteristics of the FITNESS cohort and subgroup with valid ultrasound measurements.

	Total		Males		Females	
	*n*	IQR (%)	*n*	IQR (%)	*n*	IQR (%)
Male	138	61.9	—	—	—	—
Frailty	76	34.1	43	31.2	33	38.8
Albumin^*∗*^	39	35–42	40	36–42	38	34–38
MoCA^*∗*^	22	18–25	22	19–25	22	17–25
Age^*∗*^	63	54–74	63	55–72	63	53–75
BMI^*∗*^	27.0	23.4–32.1	26.9	23.9–32.1	27.3	22.7–32.4
Charlson index^*∗∗∗*^	5	3–6	5	3–6	4	3–5
HD vintage (months)^*∗*^	33	14–65	31	12–64	37	17–66
Kt/V^*∗*^	1.58	1.37–1.82	1.48	1.32–1.69	1.80	1.52–2.00

*Ethnicity*						
White	136	61.0	75	54.4	61	71.8
South Asian	51	22.9	42	30.4	9	10.6
Black	32	14.4	19	13.8	13	15.3
Other	4	1.8	2	1.5	2	2.4

*Smoking status*						
Current	33	14.8	16	11.6	17	30.0
Ex	67	30.0	48	34.8	19	22.4
Never	123	55.2	74	53.6	49	57.7

*Active on transplant list?*						
No	195	87.4	122	88.4	73	85.9
Yes	28	12.6	16	11.6	12	14.1

*Employment status*						
Employed	32	14.4	24	17.4	8	9.4
Unemployed	74	33.2	48	34.8	26	30.6
Retired	117	52.5	66	47.8	51	60.0

*Occupation* ^*∗∗∗*^						
Unskilled manual	92	43.4	61	45.9	31	39.2
Skilled manual	32	15.1	27	20.3	5	6.3
Clerical	25	11.8	6	4.5	19	24.1
Managerial	31	14.6	23	17.3	8	10.1
Professional	32	15.1	16	12	16	20.3

*Education level*						
High school	154	69.1	99	71.7	55	64.7
College/6th form	47	21.1	26	18.8	21	24.7
University	22	9.9	13	9.4	9	10.6

*Type of residence*						
Own home	219	98.2	135	97.8	84	98.8
Warden-controlled	3	1.4	3	2.2	0	0
Residential home	1	0.5	0	0	1	1.2

*Professional carer use?* ^*∗∗∗∗*^						
No	209	94.1	130	94.2	79	94.1
Yes	13	5.9	8	5.8	5	6.0

*Physical activity index*						
Inactive	184	82.5	108	78.3	76	89.4
Moderately inactive	18	8.1	14	10.1	4	4.7
Moderately active	5	2.2	2	1.5	3	3.5
Active	16	7.2	14	10.1	2	2.4

*IMD quintile*						
1	101	45.3	64	46.4	37	43.5
2	29	13.0	16	11.6	13	15.3
3	36	16.1	26	18.8	10	11.8
4	19	8.5	9	6.5	10	11.8
5	22	9.9	13	9.4	9	10.6
Unknown	16	7.2	10	7.3	6	7.1

All values *n* and percentages except ^*∗*^ = median and interquartile range. Frailty assessed by frailty phenotype. ^*∗∗*^ = CKD omitted; ^*∗∗∗*^ = or previous occupation if unemployed/retired; ^*∗∗∗∗*^= if not in residential/nursing accommodation.

**Table 2:** Prevalence of low muscle mass, low muscle function, and sarcopenia by gender.

	Male	Female	*P*
*Low muscle mass*			
No	**50**	**54**	**<0.01**
	**36.2%**	**63.5%**	
Yes	**88**	**31**	
	**63.8%**	**36.5%**	

*Sarcopenia*			
No	88	64	0.07
	63.8%	75.3%	
Yes	50	21	
	36.2%	24.7%	

*Severe sarcopenia*			
No	104	70	0.22
	75.4%	82.4%	
Yes	34	15	
	24.6%	17.7%	

*Low grip strength*			
No	78	46	0.73
	56.5%	54.1%	
Yes	60	39	
	43.5%	45.9%	

*Slow walk speed*			
No	78	40	0.17
	56.5%	47.1%	
Yes	60	45	
	43.5%	52.9%	

Percentages within gender shown. *P* values obtained by the Chi-squared test. Bold values indicate significance at *P* < 0.05.

**Table 3:** Proportions of low muscle mass, low grip strength, slow walking speed, sarcopenia, and severe sarcopenia by gender.

	Male (*n* = 138)		Female (*n* = 85)		*P*
	*n*	%	*n*	%	
*Low muscle mass*					
18–34	2	50	2	29	0.58
35–44	4	50	1	20	0.57
45–54	9	43	5	42	0.95
55–64	**27**	**64**	**6**	**29**	**<0.01**
≥65	**46**	**73**	**17**	**43**	**<0.01**

*Low grip strength*					
18–34	0	0	3	43	0.24
35–44	1	13	3	60	0.22
45–54	5	24	4	33	0.69
55–64	24	57	7	33	0.08
≥65	30	48	22	55	0.47

*Slow walking speed*					
18–34	0	0	2	29	0.24
35–44	2	25	3	60	0.29
45–54	6	29	5	42	0.47
55–64	17	40	10	48	0.59
≥65	35	56	25	63	0.49

*Sarcopenia*					
18–34	0	0	2	29	0.24
35–44	0	0	1	20	0.19
45–54	5	23	3	25	0.94
55–64	**19**	**45**	**4**	**19**	**0.04**
≥65	26	41	11	28	0.16

*Severe sarcopenia*					
18–34	0	0	1	14	1.00
35–44	0	0	1	20	0.39
45–54	2	10	2	17	0.61
55–64	10	24	2	10	0.31
≥65	22	35	9	23	0.18

*n* = number of participants within age and gender groups with attribute. % = percentage of participants within the age and gender groups with attribute. Bold text indicates significance at <0.05 level.

**Table 4:** Proportions of low muscle mass, low grip strength, slow walking speed, sarcopenia, and severe sarcopenia by age tercile.

	Male (*n* = 138)		Female (*n* = 85)		*P*
	*n*	%	*n*	%	
*Low muscle mass*					
<57	19	45	8	30	0.20
57–70	**39**	**72**	**9**	**32**	**<0.01**
>70	**30**	**71**	**14**	**47**	**0.03**

*Low grip strength*					
<57	12	29	10	37	0.46
57–70	27	50	11	39	0.36
>70	21	50	18	60	0.40

*Slow walking speed*					
<57	11	26	10	37	0.34
57–70	25	46	16	57	0.35
>70	24	57	19	63	0.60

*Sarcopenia*					
<57	9	21	6	22	0.94
57–70	23	43	6	21	0.057
>70	18	43	9	30	0.27

*Severe sarcopenia*					
<57	3	71	4	15	0.42
57–70	16	30	4	14	0.18
>70	15	36	7	23	0.26

All definitions by EQGSOP guidelines. *P* values for difference obtained via chi-squared or Fisher's Exact as appropriate. Bold text indicates significance at *P* < 0.05 level. *n* = number of participants within age and gender groups with attribute. % = percentage of participants within the age and gender groups with attribute.

**Table 5:** Multiple linear regressions of grip strength and walking speed by low muscle mass.

		β	Lower 95% C.I.	Upper 95% C.I.	*P*
Low muscle mass	*Grip strength*				
	All	**−3.23**	**−5.65**	**−0.82**	**<0.01**
	Males	**−4.17**	**−7.57**	**−0.77**	**0.02**
	Females	−1.88	−5.41	1.64	0.29
	*Walking speed*				
	All	**−0.115**	**−0.202**	**−0.028**	**0.01**
	Males	**−0.136**	**−0.258**	**−0.013**	**0.03**
	Females	**−0.152**	**−0.300**	**−0.005**	**0.04**

Grip strength in kg. Walking speed in ms^−1^. Bold text indicates significance at *P* < 0.05 level. Multiple linear regression adjusted for BMI, ultrafiltration volume remaining at time of scan (ml/kg dry weight), ethnicity, age, IMD quintile, Charlson index, self-reported health change, physical activity index (PAI) derived from GP physical activity questionnaire, [31, 32] use of walking aids (yes/no), EQVAS, and haemodialysis vintage (months).

**Table 6:** Comparison of constituents of the frailty phenotype by gender.

	Males		Females		*P*
	*n*	%	*n*	%	
*Whole cohort (n* *=* *485)*					
Slow walking speed	**102**	**35.92**	**104**	**51.74**	**<0.01**
Weak grip	184	64.79	134	66.67	0.67
Weight loss	36	12.68	25	12.44	0.94
Exhaustion	131	46.13	106	52.74	0.15
Low energy expenditure	**123**	**43.31**	**109**	**54.23**	**0.02**

*With valid US (n* *=* *223)*					
Slow walking speed	**42**	**30.43**	**37**	**43.53**	**0.05**
Weak grip	86	62.32	51	60.00	0.73
Weight loss	17	12.32	8	9.41	0.50
Exhaustion	62	44.93	47	55.29	0.13
Low energy expenditure	54	39.13	35	41.18	0.76

*Without valid US (n* *=* *262)*					
Slow walking speed	**60**	**41.10**	**67**	**57.76**	**<0.01**
Weak grip	98	67.12	83	71.55	0.44
Weight loss	19	13.01	17	14.66	0.70
Exhaustion	69	47.26	59	50.86	0.56
Low energy expenditure	**69**	**47.26**	**74**	**63.79**	**<0.01**

Whole FITNESS cohort and those with and without ultrasound measurements are shown. *P* values are obtained by Chi-squared. % = within group percentage. *n* = number of participants within age and gender groups with attribute. % = percentage of participants within the age and gender groups with attribute. Bold text indicates significance at *P* < 0.05 level.

## Data Availability

The data used to support the study are available from the corresponding author upon request.

## Supplementary Materials

Supplementary Materials Supplementary 1. Supplementary Methods. Description of how frailty, vulnerability, and robustness defined. Supplementary 2. Supplementary Table 1: Frailty Phenotype. Supplementary 3. Supplementary Table 2: Prevalence of FP frailty, low grip strength, and slow walking speed stratified by availability of ultrasound data and gender. Supplementary 4. Supplementary Table 3: Multiple linear regression model of grip strength associated with BATT in males. Supplementary 5. Supplementary Table 4: Multiple linear regression model of grip strength associated with BATT in females. Supplementary 6. Supplementary Table 5: Multiple linear regression model of walking speed associated with BATT in males. Supplementary 7. Supplementary Table 6: Multiple linear regression model of walking speed associated with BATT in females. Supplementary 8. Supplementary Table 7a: Simple Linear Regression of Frailty Phenotype score associated with BATT in males. Supplementary 9. Supplementary Table 7b: Multiple Linear Regression of Frailty Phenotype scores associated with BATT in males. Model 1. Supplementary 10. Supplementary Table 7c: Multiple Linear Regression of Frailty Phenotype scores associated with BATT in males. Model 2. Supplementary 11. Supplementary Table 7d: Multiple Linear Regression of Frailty Phenotype scores associated with BATT in males. Model 3. Supplementary 12. Supplementary Table 8a: Simple Linear Regression of Frailty Phenotype score associated with BATT in females. Supplementary 13. Supplementary Table 8b: Multiple Linear Regression of Frailty Phenotype scores associated with BATT in females. Model 1. Supplementary 14. Supplementary Table 8c: Multiple Linear Regression of Frailty Phenotype scores associated with BATT in females. Model 2. Supplementary 15. Supplementary Table 8d: Multiple Linear Regression of Frailty Phenotype scores associated with BATT in females. Model 3. Supplementary 16. Supplementary Table 9a: Simple linear regression of Frailty Phenotype score by LMM in males. Supplementary 17. Supplementary Table 9b: Multiple linear regression of Frailty Phenotype score by LMM in males. Model 1. Supplementary 18. Supplementary Table 9c: Multiple linear regression of Frailty Phenotype score by LMM in males. Model 2. Supplementary 19. Supplementary Table 9d: Multiple linear regression of Frailty Phenotype score by LMM in males. Model 3. Supplementary 20. Supplementary Table 10a: Simple linear regression of frailty phenotype score by LMM in females. Supplementary 21. Supplementary Table 10b: Multiple linear regression of Frailty Phenotype score by LMM in females. Model 1. Supplementary 22. Supplementary Table 10c: Multiple linear regression of Frailty Phenotype score by LMM in females. Model 2. Supplementary 23. Supplementary Table 10d: Multiple linear regression of Frailty Phenotype score by LMM in females. Model 3. Supplementary 24. Supplementary Table 11a: Simple linear regression of frailty phenotype score by sarcopenia (EWGSOP definition) in males. Supplementary 25. Supplementary Table 11b: Multiple linear regression of frailty phenotype score by sarcopenia (EWGSOP definition) in males. Model 1. Supplementary 26. Supplementary Table 11c: Multiple linear regression of frailty phenotype score by sarcopenia (EWGSOP definition) in males. Model 2. Supplementary 27. Supplementary Table 11d: Multiple linear regression of frailty phenotype score by sarcopenia (EWGSOP definition) in males. Model 3. Supplementary 28. Supplementary Table 12a: Simple linear regression of frailty phenotype score by sarcopenia (EWGSOP definition) in females. Supplementary 29. Supplementary Table 12b: Multiple linear regression of frailty phenotype score by sarcopenia (EWGSOP definition) in females. Model 1. Supplementary 30. Supplementary Table 12c: Multiple linear regression of frailty phenotype score by sarcopenia (EWGSOP definition) in females. Model 2. Supplementary 31. Supplementary Table 12d: Multiple linear regression of frailty phenotype score by sarcopenia (EWGSOP definition) in females. Model 3. Supplementary 32. Supplementary Table 13a: Logistic Regression of FP frailty by BATT in males. Univariable. Supplementary 33. Supplementary Table 13b: Multivariable Logistic Regression of FP frailty by BATT in males. Model 1. Supplementary 34. Supplementary Table 13c: Multivariable Logistic Regression of FP frailty by BATT in males. Model 2. Supplementary 35. Supplementary Table 13d: Multivariable Logistic Regression of FP frailty by BATT in males. Model 3. Supplementary 36. Supplementary Table 14a: Logistic Regression of FP frailty by BATT in females. Univariable. Supplementary 37. Supplementary Table 14b: Multivariable Logistic Regression of FP frailty by BATT in females. Model 1. Supplementary 38. Supplementary Table 14c: Multivariable Logistic Regression of FP frailty by BATT in females. Model 2. Supplementary 39. Supplementary Table 14d: Multivariable Logistic Regression of FP frailty by BATT in females. Model 3. Supplementary 40. Supplementary Table 15a: Univariable logistic regression of FP frailty by low muscle mass in males. Supplementary 41. Supplementary Table 15b: Multivariable logistic regression of FP frailty by low muscle mass in males. Model 1. Supplementary 42. Supplementary Table 15c: Multivariable logistic regression of FP frailty by low muscle mass in males. Model 2. Supplementary 43. Supplementary Table 15d: Multivariable logistic regression of FP frailty by low muscle mass in males. Model 3. Supplementary 44. Supplementary Table 16a: Univariable logistic regression of FP frailty by low muscle mass in females. Supplementary 45. Supplementary Table 16b: Multivariable logistic regression of FP frailty by low muscle mass in females. Model 1. Supplementary 46. Supplementary Table 16c: Multivariable logistic regression of FP frailty by low muscle mass in females. Model 2. Supplementary 47. Supplementary Table 16d: Multivariable logistic regression of FP frailty by low muscle mass in females. Model 3. Supplementary 48. Supplementary Table 17a: Univariable logistic regression of FP frailty by sarcopenia (EWGSOP definition) in males. Supplementary 49. Supplementary Table 17b: Multivariable logistic regression of FP frailty by sarcopenia (EWGSOP definition) in males. Model 1. Supplementary 50. Supplementary Table 17c: Multivariable logistic regression of FP frailty by sarcopenia (EWGSOP definition) in males. Model 2. Supplementary 51. Supplementary Table 17d: Multivariable logistic regression of FP frailty by sarcopenia (EWGSOP definition) in males. Model 3. Supplementary 52. Supplementary Table 18a: Univariable logistic regression of FP frailty by sarcopenia (EWGSOP definition) in females. Supplementary 53. Supplementary Table 18b: Multivariable logistic regression of FP frailty by sarcopenia (EWGSOP definition) in females. Model 1. Supplementary 54. Supplementary Table 18c: Multivariable logistic regression of FP frailty by sarcopenia (EWGSOP definition) in females. Model 2. Supplementary 55. Supplementary Table 18d: Multivariable logistic regression of FP frailty by sarcopenia (EWGSOP definition) in females. Model 3.
