# Supplementary material for: Gender Disparity in Expression of Sarcopenia in Haemodialysis Recipients: Analysis from the FITNESS Cohort
Source: Int J Nephrol. 2023 Jun 17;2023:5885059. doi: 10.1155/2023/5885059 (PMC10290558; doi:10.1155/2023/5885059)
Supplement: Supplementary Materials — Supplementary 1. Supplementary Methods. Description of how frailty, vulnerability, and robustness defined. Supplementary 2. Supplementary Table 1: Frailty Phenotype. Supplementary 3. Supplementary Table 2: Prevalence of FP frailty, low grip strength, and slow walking speed stratified by availability of ultrasound data and gender. Supplementary 4. Supplementary Table 3: Multiple linear regression model of grip strength associated with BATT in males. Supplementary 5. Supplementary Table 4: Multiple linear regression model of grip strength associated with BATT in females. Supplementary 6. Supplementary Table 5: Multiple linear regression model of walking speed associated with BATT in males. Supplementary 7. Supplementary Table 6: Multiple linear regression model of walking speed associated with BATT in females. Supplementary 8. Supplementary Table 7a: Simple Linear Regression of Frailty Phenotype score associated with BATT in males. Supplementary 9. Supplementary Table 7b: Multiple Linear Regression of Frailty Phenotype scores associated with BATT in males. Model 1. Supplementary 10. Supplementary Table 7c: Multiple Linear Regression of Frailty Phenotype scores associated with BATT in males. Model 2. Supplementary 11. Supplementary Table 7d: Multiple Linear Regression of Frailty Phenotype scores associated with BATT in males. Model 3. Supplementary 12. Supplementary Table 8a: Simple Linear Regression of Frailty Phenotype score associated with BATT in females. Supplementary 13. Supplementary Table 8b: Multiple Linear Regression of Frailty Phenotype scores associated with BATT in females. Model 1. Supplementary 14. Supplementary Table 8c: Multiple Linear Regression of Frailty Phenotype scores associated with BATT in females. Model 2. Supplementary 15. Supplementary Table 8d: Multiple Linear Regression of Frailty Phenotype scores associated with BATT in females. Model 3. Supplementary 16. Supplementary Table 9a: Simple linear regression of Frailty Phenotype score by LMM i [file 5885059.f1.docx]

**Supplementary Methods. Description of how frailty, vulnerability and robustness defined**

The Frailty Phenotype was determined as a score between zero and five, with participants receiving one point for each of the following: slow walking speed, weak grip strength, exhaustion, weight loss, and low physical activity. Slow walking speed was assessed from a standing start over 4 metres, and participants scored one point if their time was over or equal to prespecified values stratified by height and gender or if they were unable to undertake the test. These values were determined by the original FP described by Fried and colleagues^1^, with times reduced in proportion to the reduced distance of 4m in this study versus 15 feet in the FP study. Poor grip strength was evaluated with dynamometer (Takei Grip D, Takei Scientific Instruments, Niigata, Japan); participants scored one point if their grip strength in either hand was not above cut-off values as per the original FP^1^ stratified by BMI and gender, or if they were unable to perform the test in either hand. Unintentional weight loss of greater than 4.5kg in the past year scored one point. To characterise exhaustion, participants were asked whether they felt that a) everything was an effort or b) they felt they could not get going within the past week. The possible responses were “Rarely”, “Some of the time”, and “Most of the time”. Participants scored one point for exhaustion if they responded that they felt either statement was true either most of the time or - if they answered “Some of the time” – they felt it was the case on more than 3 days in the week prior to recruitment. Physical activity was assessed by response to the question “How often do you engage in activities that require a low or moderate level of energy such as gardening, cleaning the car, or going for a walk?”. Possible responses were “More than once per week”, “Once per week”, “One to three times per month”, and “Hardly ever or never”. Participants scored one point for low physical activity if they responded “One to three times per month” or “Hardly ever or never”. Participants were considered not frail by FP if their total score was 0, vulnerable if their total score was 1-2 and frail if their score was 3-5.

Supplementary Table 1: Frailty Phenotype

**
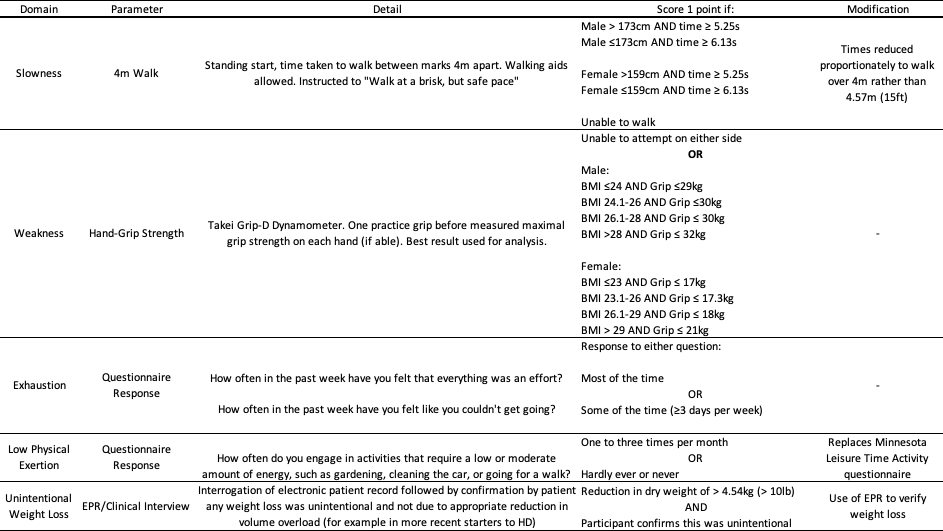
**

Supplementary Table 2: Prevalence of FP frailty, low grip strength, and slow walking speed stratified by availability of ultrasound data and gender

|  | Without US (n=262) | | With USS (n=223) | | P |
| --- | --- | --- | --- | --- | --- |
|  | n | % | n | % |  |
| Combined |  |  |  |  |  |
| *Frail* | **127** | **48%** | **76** | **34%** | **0.001** |
| *Low Grip Strength* | **143** | **55%** | **99** | **44%** | **0.025** |
| *Slow Walking Speed* | **158** | **60%** | **105** | **47%** | **0.004** |
| Males |  |  |  |  |  |
| *Frail* | **63** | **43%** | **43** | **31%** | **0.037** |
| *Low Grip Strength* | 76 | 52% | 60 | 43% | 0.148 |
| *Slow Walking Speed* | 79 | 54% | 60 | 43% | 0.073 |
| Females |  |  |  |  |  |
| *Frail* | **64** | **55%** | **33** | **39%** | **0.022** |
| *Low Grip Strength* | 67 | 58% | 39 | 46% | 0.096 |
| *Slow Walking Speed* | **79** | **68%** | **45** | **53%** | **0.029** |

% = prevalence within group. P value for difference obtained via Chi-squared.

Supplementary Table 3: Multiple linear regression model of grip strength associated with BATT in males.

|  | Coefficient | Lower 95% C.I. | Upper 95% C.I. | P |
| --- | --- | --- | --- | --- |
| BATT | **0.13** | **0.01** | **0.24** | **0.030** |
| BMI | 0.09 | -0.17 | 0.35 | 0.501 |
| UF (ml/kg)* | -0.08 | -0.21 | 0.06 | 0.272 |
| Ethnicity |  |  |  |  |
| *White* | REFERENCE | | | |
| *South Asian* | -2.95 | -6.31 | 0.41 | 0.085 |
| *Black* | 2.82 | -1.62 | 7.27 | 0.210 |
| *Other* | **12.1** | **0.22** | **23.91** | **0.046** |
| Age | -0.13 | -0.29 | 0.03 | 0.111 |
| IMD Quintile | |  |  |  |
| *1* | REFERENCE | | | |
| *2* | 1.91 | -2.61 | 6.43 | 0.404 |
| *3* | 2.47 | -1.48 | 6.43 | 0.218 |
| *4* | -2.30 | -8.56 | 3.96 | 0.468 |
| *5* | 1.24 | -4.02 | 6.49 | 0.642 |
| *Unknown* | 1.57 | -4.07 | 7.21 | 0.581 |
| Charlson Index** | -0.14 | -0.94 | 0.67 | 0.735 |
| Health Change | |  |  |  |
| *Better* | REFERENCE | | | |
| *The Same* | -2.06 | -6.10 | 1.98 | 0.314 |
| *Worse* | -0.91 | -4.72 | 2.90 | 0.636 |
| Physical Activity Index | |  |  |  |
| *Inactive* | REFERENCE | | | |
| *Moderately Inactive* | 1.72 | -3.10 | 6.54 | 0.481 |
| *Moderately Active* | 3.42 | -8.09 | 14.94 | 0.557 |
| *Active* | 3.16 | -1.65 | 7.97 | 0.196 |
| Walking Aid Use | |  |  |  |
| *No* | REFERENCE | | | |
| *Yes* | -2.81 | -6.08 | 0.46 | 0.091 |
| EQ-5D Health Today | 0.04 | -0.03 | 0.11 | 0.271 |
| HD Vintage | -0.02 | -0.05 | 0.00 | 0.092 |
| Constant | **28.9** | **15.3** | **42.5** | **<0.001** |

Bold text indicates significance at P<0.005 level. *=Remaining fluid removal via ultrafiltration to be completed at time of ultrasound assessment. **=CKD omitted.

Supplementary Table 4: Multiple linear regression model of grip strength associated with BATT in females.

|  | Coefficient | Lower 95% C.I. | Upper 95% C.I. | P |
| --- | --- | --- | --- | --- |
| BATT | 0.03 | -0.10 | 0.15 | 0.655 |
| BMI | 0.29 | -0.01 | 0.58 | 0.056 |
| UF (ml/kg)* | -0.01 | -0.23 | 0.22 | 0.953 |
| Ethnicity |  |  |  |  |
| *White* | REFERENCE | | | |
| *South Asian* | -4.89 | -10.05 | 0.26 | 0.062 |
| *Black* | 1.12 | -3.31 | 5.55 | 0.614 |
| *Other* | -0.95 | -12.2 | 10.31 | 0.866 |
| Age | 0.00 | -0.13 | 0.13 | 0.973 |
| IMD Quintile | |  |  |  |
| *1* | REFERENCE | | | |
| *2* | -1.74 | -6.22 | 2.74 | 0.441 |
| *3* | -1.41 | -6.45 | 3.63 | 0.577 |
| *4* | 2.12 | -2.88 | 7.12 | 0.399 |
| *5* | 4.60 | -0.34 | 9.54 | 0.067 |
| *Unknown* | 3.12 | -3.17 | 9.40 | 0.325 |
| Charlson Index** | -0.67 | -1.62 | 0.29 | 0.166 |
| Health Change | |  |  |  |
| *Better* | REFERENCE | | | |
| *The Same* | 0.93 | -3.73 | 5.60 | 0.691 |
| *Worse* | 0.73 | -3.90 | 5.35 | 0.755 |
| Physical Activity Index | |  |  |  |
| *Inactive* | REFERENCE | | | |
| *Moderately Inactive* | **9.98** | **2.33** | **17.63** | **0.011** |
| *Moderately Active* | 0.67 | -7.27 | 8.61 | 0.867 |
| *Active* | 0.58 | -10.06 | 11.22 | 0.913 |
| Walking Aid Use | |  |  |  |
| *No* | REFERENCE | | | |
| *Yes* | **-3.66** | **-7.05** | **-0.27** | **0.035** |
| EQ-5D Health Today | 0.03 | -0.04 | 0.10 | 0.407 |
| HD Vintage | 0.00 | -0.03 | 0.03 | 0.980 |
| Constant | 9.11 | -3.86 | 22.1 | 0.165 |

Bold text indicates significance at P<0.005 level. *=Remaining fluid removal via ultrafiltration to be completed at time of ultrasound assessment. **=CKD omitted.

Supplementary Table 5: Multiple linear regression model of walking speed associated with BATT in males.

|  | Coefficient | Lower 95% C.I. | Upper 95% C.I. | P |
| --- | --- | --- | --- | --- |
| BATT | **0.006** | **0.002** | **0.010** | **0.004** |
| BMI | **-0.013** | **-0.023** | **-0.004** | **0.006** |
| UF (ml/kg)* | -0.003 | -0.008 | 0.002 | 0.193 |
| Ethnicity |  |  |  |  |
| *White* | REFERENCE | | | |
| *South Asian* | **-0.127** | **-0.241** | **-0.013** | **0.029** |
| *Black* | -0.015 | -0.168 | 0.138 | 0.845 |
| *Other* | 0.140 | -0.245 | 0.524 | 0.473 |
| Age | 0.000 | -0.006 | 0.005 | 0.962 |
| IMD Quintile |  |  |  |  |
| *1* | REFERENCE | | | |
| *2* | 0.089 | -0.062 | 0.241 | 0.244 |
| *3* | 0.099 | -0.035 | 0.233 | 0.145 |
| *4* | 0.154 | -0.062 | 0.370 | 0.160 |
| *5* | 0.041 | -0.145 | 0.227 | 0.663 |
| *Unknown* | -0.060 | -0.245 | 0.124 | 0.520 |
| Charlson Index** | -0.028 | -0.056 | 0.000 | 0.050 |
| Health Change |  |  |  |  |
| *Better* | REFERENCE | | | |
| *The Same* | -0.108 | -0.243 | 0.028 | 0.118 |
| *Worse* | -0.049 | -0.179 | 0.080 | 0.454 |
| Physical Activity Index |  |  |  |  |
| *Inactive* | REFERENCE | | | |
| *Moderately Inactive* | 0.149 | -0.013 | 0.311 | 0.070 |
| *Moderately Active* | 0.117 | -0.257 | 0.491 | 0.535 |
| *Active* | **0.214** | **0.057** | **0.372** | **0.008** |
| Walking Aid Use |  |  |  |  |
| *No* | REFERENCE | | | |
| *Yes* | **-0.197** | **-0.309** | **-0.084** | **0.001** |
| EQ-5D Health Today | 0.002 | 0.000 | 0.004 | 0.107 |
| HD Vintage | 0.000 | -0.001 | 0.000 | 0.339 |
| Constant | **1.170** | **0.709** | **1.632** | **<0.001** |

Bold text indicates significance at P<0.005 level. *=Remaining fluid removal via ultrafiltration to be completed at time of ultrasound assessment. **=CKD omitted.

Supplementary Table 6: Multiple linear regression model of walking speed associated with BATT in females.

|  | Coefficient | Lower 95% C.I. | Upper 95% C.I. | P |
| --- | --- | --- | --- | --- |
| BATT | 0.003 | -0.002 | 0.008 | 0.269 |
| BMI | -0.009 | -0.023 | 0.005 | 0.187 |
| UF (ml/kg)* | 0.000 | -0.009 | 0.009 | 0.946 |
| Ethnicity |  |  |  |  |
| *White* | REFERENCE | | | |
| *South Asian* | -0.063 | -0.287 | 0.160 | 0.574 |
| *Black* | -0.018 | -0.186 | 0.150 | 0.830 |
| *Other* | 0.260 | -0.167 | 0.686 | 0.227 |
| Age | -0.002 | -0.008 | 0.004 | 0.470 |
| IMD Quintile |  |  |  |  |
| *1* | REFERENCE | | | |
| *2* | -0.043 | -0.222 | 0.136 | 0.632 |
| *3* | 0.083 | -0.102 | 0.269 | 0.373 |
| *4* | 0.115 | -0.069 | 0.300 | 0.214 |
| *5* | 0.072 | -0.114 | 0.259 | 0.441 |
| *Unknown* | -0.095 | -0.365 | 0.174 | 0.481 |
| Charlson Index** | -0.005 | -0.045 | 0.034 | 0.790 |
| Health Change |  |  |  |  |
| *Better* | REFERENCE | | | |
| *The Same* | -0.028 | -0.215 | 0.159 | 0.763 |
| *Worse* | -0.008 | -0.192 | 0.176 | 0.929 |
| Physical Activity Index |  |  |  |  |
| *Inactive* | REFERENCE | | | |
| *Moderately Inactive* | **0.382** | **0.088** | **0.676** | **0.012** |
| *Moderately Active* | -0.064 | -0.356 | 0.228 | 0.663 |
| *Active* | 0.014 | -0.377 | 0.405 | 0.942 |
| Walking Aid Use |  |  |  |  |
| *No* | REFERENCE | | | |
| *Yes* | **-0.235** | **-0.362** | **-0.109** | **<0.001** |
| EQ-5D Health Today | 0.001 | -0.002 | 0.004 | 0.465 |
| HD Vintage | -0.001 | -0.002 | 0.001 | 0.395 |
| Constant | **1.133** | **0.599** | **1.668** | **<0.001** |

Bold text indicates significance at P<0.005 level. *=Remaining fluid removal via ultrafiltration to be completed at time of ultrasound assessment. **=CKD omitted.

Supplementary Table 7a: Simple Linear Regression of Frailty Phenotype score associated with BATT in males.

|  | Coefficient | Lower 95% C.I. | Upper 95% C.I. | P |
| --- | --- | --- | --- | --- |
| BATT | -0.034 | -0.049 | -0.019 | <0.001 |
| Constant | 3.628 | 2.840 | 4.415 | <0.001 |

Supplementary Table 7b: Multiple Linear Regression of Frailty Phenotype scores associated with BATT in males. Model 1

|  | Coefficient | Lower 95% C.I. | Upper 95% C.I. | P |
| --- | --- | --- | --- | --- |
| BATT | -0.031 | -0.047 | -0.015 | <0.001 |
| Age | 0.020 | 0.001 | 0.038 | 0.038 |
| Ethnicity |  |  |  |  |
| *White* | REFERENCE | | | |
| *South Asian* | 0.325 | -0.212 | 0.862 | 0.233 |
| *Black* | -0.351 | -1.052 | 0.350 | 0.323 |
| *Other* | -0.789 | -2.814 | 1.236 | 0.442 |
| Education Level |  |  |  |  |
| *High School* | REFERENCE | | | |
| *College/6th form* | -0.121 | -0.687 | 0.445 | 0.673 |
| *University* | -0.684 | -1.511 | 0.142 | 0.104 |
| IMD Quintile |  |  |  |  |
| *1* | REFERENCE | | | |
| *2* | -0.577 | -1.284 | 0.130 | 0.109 |
| *3* | -0.416 | -1.048 | 0.217 | 0.196 |
| *4* | 0.028 | -0.925 | 0.981 | 0.954 |
| *5* | -0.679 | -1.498 | 0.140 | 0.103 |
| *Unknown* | -0.770 | -1.646 | 0.107 | 0.085 |
| Social Support |  |  |  |  |
| *Yes* | REFERENCE | | | |
| *No* | 0.089 | -0.845 | 1.023 | 0.851 |
| HD vintage (months) | -0.002 | -0.006 | 0.002 | 0.420 |
| Constant | 2.610 | 0.906 | 4.313 | 0.003 |

Supplementary Table 7c: Multiple Linear Regression of Frailty Phenotype scores associated with BATT in males. Model 2

|  | Coefficient | Lower 95% C.I. | Upper 95% C.I. | P |
| --- | --- | --- | --- | --- |
| BATT | -0.020 | -0.034 | -0.007 | 0.003 |
| Age | 0.019 | -0.002 | 0.040 | 0.078 |
| Ethnicity |  |  |  |  |
| *White* | REFERENCE | | | |
| *South Asian* | 0.424 | -0.075 | 0.922 | 0.095 |
| *Black* | -0.258 | -0.829 | 0.312 | 0.372 |
| *Other* | -1.376 | -3.028 | 0.276 | 0.101 |
| Education Level |  |  |  |  |
| *High School* | REFERENCE | | | |
| *College/6th form* | -0.033 | -0.515 | 0.449 | 0.892 |
| *University* | -0.344 | -1.053 | 0.365 | 0.338 |
| IMD Quintile |  |  |  |  |
| *1* | REFERENCE | | | |
| *2* | -0.005 | -0.629 | 0.619 | 0.987 |
| *3* | -0.094 | -0.618 | 0.430 | 0.722 |
| *4* | -0.187 | -0.996 | 0.621 | 0.646 |
| *5* | -0.210 | -0.908 | 0.488 | 0.552 |
| *Unknown* | -0.139 | -0.930 | 0.651 | 0.727 |
| Social Support |  |  |  |  |
| *Yes* | REFERENCE | | | |
| *No* | -0.354 | -1.170 | 0.462 | 0.392 |
| HD vintage (months) | -0.002 | -0.005 | 0.002 | 0.377 |
| Charlson Index* | 0.063 | -0.046 | 0.172 | 0.257 |
| Cognitive Impairment |  |  |  |  |
| *No* | REFERENCE | | | |
| *Yes* | -0.081 | -0.614 | 0.452 | 0.763 |
| PHQ-9 score | 0.075 | 0.033 | 0.117 | 0.001 |
| Self-reported health change |  |  |  |  |
| *Better* | REFERENCE | | | |
| *The Same* | -0.058 | -0.598 | 0.483 | 0.832 |
| *Worse* | 0.245 | -0.272 | 0.762 | 0.350 |
| Self-reported health today/100 | -0.021 | -0.031 | -0.012 | <0.001 |
| Constant | 2.370 | 0.728 | 4.013 | 0.005 |

Supplementary Table 7d: Multiple Linear Regression of Frailty Phenotype scores associated with BATT in males. Model 3

|  | Coefficient | Lower 95% C.I. | Upper 95% C.I. | P |
| --- | --- | --- | --- | --- |
| BATT | -0.015 | -0.028 | -0.003 | 0.018 |
| Age | 0.005 | -0.015 | 0.025 | 0.620 |
| Ethnicity |  |  |  |  |
| *White* | REFERENCE | | | |
| *South Asian* | 0.454 | 0.006 | 0.901 | 0.047 |
| *Black* | -0.111 | -0.644 | 0.422 | 0.680 |
| *Other* | -1.633 | -3.122 | -0.145 | 0.032 |
| Education Level |  |  |  |  |
| *High School* | REFERENCE | | | |
| *College/6th form* | -0.198 | -0.648 | 0.253 | 0.387 |
| *University* | 0.064 | -0.594 | 0.723 | 0.847 |
| IMD Quintile |  |  |  |  |
| *1* | REFERENCE | | | |
| *2* | 0.033 | -0.531 | 0.597 | 0.908 |
| *3* | 0.053 | -0.422 | 0.528 | 0.826 |
| *4* | 0.027 | -0.705 | 0.759 | 0.942 |
| *5* | -0.077 | -0.709 | 0.554 | 0.809 |
| *Unknown* | 0.045 | -0.670 | 0.761 | 0.900 |
| Social Support |  |  |  |  |
| *Yes* | REFERENCE | | | |
| *No* | -0.432 | -1.172 | 0.308 | 0.250 |
| HD vintage (months) | 0.000 | -0.003 | 0.003 | 0.969 |
| Charlson Index* | 0.067 | -0.031 | 0.165 | 0.178 |
| Cognitive Impairment |  |  |  |  |
| *No* | REFERENCE | | | |
| *Yes* | -0.234 | -0.718 | 0.250 | 0.340 |
| PHQ-9 score | 0.068 | 0.029 | 0.106 | 0.001 |
| Self-reported health change |  |  |  |  |
| *Better* | REFERENCE | | | |
| *The Same* | -0.119 | -0.606 | 0.368 | 0.630 |
| *Worse* | 0.062 | -0.408 | 0.532 | 0.793 |
| Self-reported health today/100 | -0.013 | -0.023 | -0.004 | 0.007 |
| Walking Aid Use |  |  |  |  |
| *No* | REFERENCE | | | |
| *Yes* | 0.653 | 0.233 | 1.074 | 0.003 |
| Self-reported slow walking |  |  |  |  |
| *No* | REFERENCE | | | |
| *Yes* | 0.697 | 0.268 | 1.126 | 0.002 |
| Constant | 1.884 | 0.391 | 3.376 | 0.014 |

Supplementary Table 8a: Simple Linear Regression of Frailty Phenotype score associated with BATT in females.

|  | Coefficient | Lower 95% C.I. | Upper 95% C.I. | P |
| --- | --- | --- | --- | --- |
| BATT | -0.013 | -0.033 | 0.007 | 0.206 |
| Constant | 2.684 | 1.721 | 3.647 | <0.001 |

Supplementary Table 8b: Multiple Linear Regression of Frailty Phenotype scores associated with BATT in females. Model 1

|  | Coefficient | Lower 95% C.I. | Upper 95% C.I. | P |
| --- | --- | --- | --- | --- |
| BATT | -0.007 | -0.030 | 0.015 | 0.501 |
| Age | 0.008 | -0.012 | 0.029 | 0.432 |
| Ethnicity |  |  |  |  |
| *White* | REFERENCE | | | |
| *South Asian* | 0.237 | -0.711 | 1.185 | 0.619 |
| *Black* | -0.494 | -1.360 | 0.371 | 0.259 |
| *Other* | -1.400 | -3.394 | 0.594 | 0.166 |
| Education Level |  |  |  |  |
| *High School* | REFERENCE | | | |
| *College/6th form* | 0.392 | -0.303 | 1.086 | 0.264 |
| *University* | -0.319 | -1.360 | 0.722 | 0.543 |
| IMD Quintile |  |  |  |  |
| *1* | REFERENCE | | | |
| *2* | 0.300 | -0.537 | 1.137 | 0.477 |
| *3* | -0.577 | -1.543 | 0.389 | 0.238 |
| *4* | -1.352 | -2.304 | -0.400 | 0.006 |
| *5* | -1.081 | -2.047 | -0.114 | 0.029 |
| *Unknown* | 0.472 | -0.743 | 1.686 | 0.441 |
| Social Support |  |  |  |  |
| *Yes* | REFERENCE | | | |
| *No* | 0.161 | -1.036 | 1.358 | 0.789 |
| HD vintage (months) | -0.001 | -0.008 | 0.006 | 0.768 |
| Constant | 2.256 | 0.403 | 4.109 | 0.018 |

Supplementary Table 8c: Multiple Linear Regression of Frailty Phenotype scores associated with BATT in females. Model 2

|  | Coefficient | Lower 95% C.I. | Upper 95% C.I. | P |
| --- | --- | --- | --- | --- |
| BATT | -0.011 | -0.034 | 0.013 | 0.370 |
| Age | 0.009 | -0.018 | 0.036 | 0.498 |
| Ethnicity |  |  |  |  |
| *White* | REFERENCE | | | |
| *South Asian* | 0.644 | -0.472 | 1.759 | 0.253 |
| *Black* | -0.266 | -1.128 | 0.596 | 0.539 |
| *Other* | -0.559 | -2.607 | 1.488 | 0.587 |
| Education Level |  |  |  |  |
| *High School* | REFERENCE | | | |
| *College/6th form* | 0.550 | -0.198 | 1.297 | 0.147 |
| *University* | -0.269 | -1.429 | 0.890 | 0.644 |
| IMD Quintile |  |  |  |  |
| *1* | REFERENCE | | | |
| *2* | 0.282 | -0.587 | 1.151 | 0.519 |
| *3* | -0.440 | -1.451 | 0.571 | 0.387 |
| *4* | -1.024 | -2.015 | -0.033 | 0.043 |
| *5* | -0.784 | -1.751 | 0.184 | 0.110 |
| *Unknown* | 0.335 | -0.950 | 1.620 | 0.604 |
| Social Support |  |  |  |  |
| *Yes* | REFERENCE | | | |
| *No* | 0.267 | -1.027 | 1.561 | 0.681 |
| HD vintage (months) | -0.001 | -0.008 | 0.006 | 0.771 |
| Charlson Index* | 0.081 | -0.138 | 0.299 | 0.464 |
| Cognitive Impairment |  |  |  |  |
| *No* | REFERENCE | | | |
| *Yes* | 0.314 | -0.495 | 1.124 | 0.440 |
| PHQ-9 score | 0.074 | 0.007 | 0.140 | 0.031 |
| Self-reported health change |  |  |  |  |
| *Better* | REFERENCE | | | |
| *The Same* | -0.096 | -1.064 | 0.873 | 0.844 |
| *Worse* | -0.350 | -1.327 | 0.626 | 0.476 |
| Self-reported health today/100 | -0.012 | -0.029 | 0.004 | 0.146 |
| Constant | 1.956 | -0.563 | 4.475 | 0.126 |

Supplementary Table 8d: Multiple Linear Regression of Frailty Phenotype scores associated with BATT in females. Model 3

|  | Coefficient | Lower 95% C.I. | Upper 95% C.I. | P |
| --- | --- | --- | --- | --- |
| BATT | 0.001 | -0.019 | 0.021 | 0.903 |
| Age | 0.003 | -0.020 | 0.025 | 0.797 |
| Ethnicity |  |  |  |  |
| *White* | REFERENCE | | | |
| *South Asian* | 0.292 | -0.641 | 1.225 | 0.534 |
| *Black* | -0.392 | -1.102 | 0.319 | 0.274 |
| *Other* | -0.206 | -1.912 | 1.499 | 0.810 |
| Education Level |  |  |  |  |
| *High School* | REFERENCE | | | |
| *College/6th form* | 0.265 | -0.361 | 0.890 | 0.401 |
| *University* | -0.290 | -1.249 | 0.670 | 0.548 |
| IMD Quintile |  |  |  |  |
| *1* | REFERENCE | | | |
| *2* | 0.277 | -0.439 | 0.993 | 0.442 |
| *3* | -0.155 | -0.992 | 0.683 | 0.713 |
| *4* | -0.908 | -1.726 | -0.090 | 0.030 |
| *5* | -1.029 | -1.829 | -0.229 | 0.013 |
| *Unknown* | 0.181 | -0.877 | 1.239 | 0.733 |
| Social Support |  |  |  |  |
| *Yes* | REFERENCE | | | |
| *No* | -0.073 | -1.174 | 1.028 | 0.894 |
| HD vintage (months) | -0.001 | -0.006 | 0.005 | 0.840 |
| Charlson Index* | -0.013 | -0.196 | 0.170 | 0.890 |
| Cognitive Impairment |  |  |  |  |
| *No* | REFERENCE | | | |
| *Yes* | 0.262 | -0.406 | 0.930 | 0.436 |
| PHQ-9 score | 0.039 | -0.017 | 0.095 | 0.169 |
| Self-reported health change |  |  |  |  |
| *Better* | REFERENCE | | | |
| *The Same* | -0.008 | -0.805 | 0.790 | 0.985 |
| *Worse* | -0.202 | -1.010 | 0.606 | 0.619 |
| Self-reported health today/100 | -0.006 | -0.020 | 0.008 | 0.365 |
| Walking Aid Use |  |  |  |  |
| *No* | REFERENCE | | | |
| *Yes* | 1.087 | 0.567 | 1.607 | <0.001 |
| Self-reported slow walking |  |  |  |  |
| *No* | REFERENCE | | | |
| *Yes* | 0.726 | 0.181 | 1.272 | 0.010 |
| Constant | 1.259 | -0.880 | 3.397 | 0.244 |

Supplementary Table 9a: Simple linear regression of Frailty Phenotype score by LMM in males.

|  | Coefficient | Lower 95% C.I. | Upper 95% C.I. | P |
| --- | --- | --- | --- | --- |
| Low Muscle Mass |  |  |  |  |
| *No* | REFERENCE | | | |
| *Yes* | 1.021 | 0.568 | 1.475 | <0.001 |
| Constant | 1.240 | 0.878 | 1.602 | <0.001 |

Supplementary Table 9b: Multiple linear regression of Frailty Phenotype score by LMM in males. Model 1

|  | Coefficient | Lower 95% C.I. | Upper 95% C.I. | P |
| --- | --- | --- | --- | --- |
| Low Muscle Mass |  |  |  |  |
| *No* | REFERENCE | | | |
| *Yes* | 1.022 | 0.544 | 1.500 | <0.001 |
| Age | 0.024 | 0.006 | 0.042 | 0.008 |
| Ethnicity |  |  |  |  |
| *White* | REFERENCE | | | |
| *South Asian* | 0.330 | -0.198 | 0.858 | 0.218 |
| *Black* | -0.308 | -0.999 | 0.383 | 0.379 |
| *Other* | -0.599 | -2.596 | 1.398 | 0.554 |
| Education Level |  |  |  |  |
| *High School* | REFERENCE | | | |
| *College/6th form* | -0.002 | -0.564 | 0.559 | 0.994 |
| *University* | -0.562 | -1.375 | 0.252 | 0.174 |
| IMD Quintile |  |  |  |  |
| *1* | REFERENCE | | | |
| *2* | -0.736 | -1.437 | -0.035 | 0.040 |
| *3* | -0.546 | -1.179 | 0.087 | 0.090 |
| *4* | 0.004 | -0.936 | 0.945 | 0.993 |
| *5* | -1.059 | -1.880 | -0.238 | 0.012 |
| *Unknown* | -0.889 | -1.753 | -0.024 | 0.044 |
| Social Support |  |  |  |  |
| *Yes* | REFERENCE | | | |
| *No* | 0.091 | -0.830 | 1.012 | 0.845 |
| HD vintage (months) | -0.002 | -0.006 | 0.002 | 0.332 |
| Constant | 0.190 | -0.978 | 1.358 | 0.748 |

Supplementary Table 9c: Multiple linear regression of Frailty Phenotype score by LMM in males. Model 2

|  | Coefficient | Lower 95% C.I. | Upper 95% C.I. | P |
| --- | --- | --- | --- | --- |
| Low Muscle Mass |  |  |  |  |
| *No* | REFERENCE | | | |
| *Yes* | 0.610 | 0.194 | 1.026 | 0.004 |
| Age | 0.020 | -0.001 | 0.041 | 0.059 |
| Ethnicity |  |  |  |  |
| *White* | REFERENCE | | | |
| *South Asian* | 0.433 | -0.065 | 0.932 | 0.088 |
| *Black* | -0.218 | -0.788 | 0.353 | 0.451 |
| *Other* | -1.228 | -2.888 | 0.432 | 0.145 |
| Education Level |  |  |  |  |
| *High School* | REFERENCE | | | |
| *College/6th form* | 0.051 | -0.435 | 0.537 | 0.835 |
| *University* | -0.282 | -0.988 | 0.425 | 0.431 |
| IMD Quintile |  |  |  |  |
| *1* | REFERENCE | | | |
| *2* | -0.134 | -0.763 | 0.494 | 0.673 |
| *3* | -0.167 | -0.700 | 0.366 | 0.537 |
| *4* | -0.172 | -0.983 | 0.639 | 0.675 |
| *5* | -0.428 | -1.139 | 0.282 | 0.235 |
| *Unknown* | -0.203 | -0.994 | 0.588 | 0.611 |
| Social Support |  |  |  |  |
| *Yes* | REFERENCE | | | |
| *No* | -0.349 | -1.167 | 0.469 | 0.399 |
| HD vintage (months) | -0.002 | -0.005 | 0.002 | 0.360 |
| Charlson Index* | 0.076 | -0.032 | 0.185 | 0.165 |
| Cognitive Impairment |  |  |  |  |
| *No* | REFERENCE | | | |
| *Yes* | -0.047 | -0.584 | 0.489 | 0.861 |
| PHQ-9 score | 0.073 | 0.031 | 0.115 | 0.001 |
| Self-reported health change |  |  |  |  |
| *Better* | REFERENCE | | | |
| *The Same* | -0.049 | -0.591 | 0.493 | 0.859 |
| *Worse* | 0.288 | -0.231 | 0.807 | 0.274 |
| Self-reported health today/100 | -0.021 | -0.031 | -0.011 | <0.001 |
| Constant | 0.730 | -0.695 | 2.155 | 0.312 |

Supplementary Table 9d: Multiple linear regression of Frailty Phenotype score by LMM in males. Model 3

|  | Coefficient | Lower 95% C.I. | Upper 95% C.I. | P |
| --- | --- | --- | --- | --- |
| Low Muscle Mass |  |  |  |  |
| *No* | REFERENCE | | | |
| *Yes* | 0.415 | 0.025 | 0.806 | 0.037 |
| Age | 0.006 | -0.014 | 0.026 | 0.545 |
| Ethnicity |  |  |  |  |
| *White* | REFERENCE | | | |
| *South Asian* | 0.466 | 0.016 | 0.917 | 0.042 |
| *Black* | -0.069 | -0.604 | 0.466 | 0.799 |
| *Other* | -1.538 | -3.040 | -0.035 | 0.045 |
| Education Level |  |  |  |  |
| *High School* | REFERENCE | | | |
| *College/6th form* | -0.128 | -0.589 | 0.333 | 0.584 |
| *University* | 0.111 | -0.547 | 0.770 | 0.738 |
| IMD Quintile |  |  |  |  |
| *1* | REFERENCE | | | |
| *2* | -0.054 | -0.624 | 0.516 | 0.850 |
| *3* | 0.011 | -0.475 | 0.498 | 0.963 |
| *4* | 0.036 | -0.702 | 0.774 | 0.923 |
| *5* | -0.223 | -0.870 | 0.424 | 0.495 |
| *Unknown* | 0.000 | -0.719 | 0.719 | 0.999 |
| Social Support |  |  |  |  |
| *Yes* | REFERENCE | | | |
| *No* | -0.442 | -1.186 | 0.303 | 0.242 |
| HD vintage (months) | 0.000 | -0.003 | 0.003 | 0.953 |
| Charlson Index* | 0.078 | -0.020 | 0.176 | 0.117 |
| Cognitive Impairment |  |  |  |  |
| *No* | REFERENCE | | | |
| *Yes* | -0.214 | -0.703 | 0.276 | 0.389 |
| PHQ-9 score | 0.066 | 0.027 | 0.105 | 0.001 |
| Self-reported health change |  |  |  |  |
| *Better* | REFERENCE | | | |
| *The Same* | -0.116 | -0.607 | 0.374 | 0.639 |
| *Worse* | 0.095 | -0.380 | 0.571 | 0.691 |
| Self-reported health today/100 | -0.013 | -0.022 | -0.003 | 0.010 |
| Walking Aid Use |  |  |  |  |
| *No* | REFERENCE | | | |
| *Yes* | 0.629 | 0.199 | 1.059 | 0.005 |
| Self-reported slow walking |  |  |  |  |
| *No* | REFERENCE | | | |
| *Yes* | 0.719 | 0.287 | 1.151 | 0.001 |
| Constant | 0.681 | -0.620 | 1.981 | 0.301 |

Supplementary Table 10a: Simple linear regression of frailty phenotype score by LMM in females.

|  | Coefficient | Lower 95% C.I. | Upper 95% C.I. | P |
| --- | --- | --- | --- | --- |
| Low Muscle Mass |  |  |  |  |
| *No* | REFERENCE | | | |
| *Yes* | 0.461 | -0.119 | 1.041 | 0.118 |
| Constant | 1.926 | 1.576 | 2.276 | <0.001 |

Supplementary Table 10b: Multiple linear regression of Frailty Phenotype score by LMM in females. Model 1

|  | Coefficient | Lower 95% C.I. | Upper 95% C.I. | P |
| --- | --- | --- | --- | --- |
| Low Muscle Mass |  |  |  |  |
| *No* | REFERENCE | | | |
| *Yes* | 0.277 | -0.344 | 0.899 | 0.376 |
| Age | 0.009 | -0.011 | 0.029 | 0.385 |
| Ethnicity |  |  |  |  |
| *White* | REFERENCE | | | |
| *South Asian* | 0.233 | -0.713 | 1.179 | 0.625 |
| *Black* | -0.499 | -1.353 | 0.356 | 0.248 |
| *Other* | -1.429 | -3.419 | 0.561 | 0.156 |
| Education Level |  |  |  |  |
| *High School* | REFERENCE | | | |
| *College/6th form* | 0.396 | -0.294 | 1.087 | 0.256 |
| *University* | -0.314 | -1.337 | 0.709 | 0.542 |
| IMD Quintile |  |  |  |  |
| *1* | REFERENCE | | | |
| *2* | 0.262 | -0.573 | 1.096 | 0.534 |
| *3* | -0.584 | -1.548 | 0.380 | 0.231 |
| *4* | -1.362 | -2.312 | -0.412 | 0.006 |
| *5* | -1.062 | -2.028 | -0.095 | 0.032 |
| *Unknown* | 0.494 | -0.719 | 1.707 | 0.420 |
| Social Support |  |  |  |  |
| *Yes* | REFERENCE | | | |
| *No* | 0.119 | -1.083 | 1.322 | 0.844 |
| HD vintage (months) | -0.001 | -0.008 | 0.005 | 0.685 |
| Constant | 1.798 | 0.382 | 3.215 | 0.014 |

Supplementary Table 10c: Multiple linear regression of Frailty Phenotype score by LMM in females. Model 2

|  | Coefficient | Lower 95% C.I. | Upper 95% C.I. | P |
| --- | --- | --- | --- | --- |
| Low Muscle Mass |  |  |  |  |
| *No* | REFERENCE | | | |
| *Yes* | 0.473 | -0.212 | 1.157 | 0.172 |
| Age | 0.011 | -0.016 | 0.038 | 0.415 |
| Ethnicity |  |  |  |  |
| *White* | REFERENCE | | | |
| *South Asian* | 0.779 | -0.352 | 1.910 | 0.173 |
| *Black* | -0.241 | -1.094 | 0.612 | 0.574 |
| *Other* | -0.644 | -2.674 | 1.386 | 0.528 |
| Education Level |  |  |  |  |
| *High School* | REFERENCE | | | |
| *College/6th form* | 0.581 | -0.150 | 1.311 | 0.117 |
| *University* | -0.251 | -1.351 | 0.849 | 0.650 |
| IMD Quintile |  |  |  |  |
| *1* | REFERENCE | | | |
| *2* | 0.189 | -0.680 | 1.059 | 0.665 |
| *3* | -0.430 | -1.431 | 0.572 | 0.394 |
| *4* | -1.021 | -2.003 | -0.038 | 0.042 |
| *5* | -0.775 | -1.734 | 0.184 | 0.111 |
| *Unknown* | 0.399 | -0.880 | 1.677 | 0.535 |
| Social Support |  |  |  |  |
| *Yes* | REFERENCE | | | |
| *No* | 0.225 | -1.060 | 1.510 | 0.727 |
| HD vintage (months) | -0.002 | -0.009 | 0.005 | 0.608 |
| Charlson Index* | 0.084 | -0.132 | 0.299 | 0.441 |
| Cognitive Impairment |  |  |  |  |
| *No* | REFERENCE | | | |
| *Yes* | 0.271 | -0.502 | 1.044 | 0.486 |
| PHQ-9 score | 0.078 | 0.012 | 0.144 | 0.022 |
| Self-reported health change |  |  |  |  |
| *Better* | REFERENCE | | | |
| *The Same* | -0.231 | -1.218 | 0.755 | 0.641 |
| *Worse* | -0.429 | -1.409 | 0.550 | 0.384 |
| Self-reported health today/100 | -0.011 | -0.027 | 0.006 | 0.206 |
| Constant | 1.213 | -1.264 | 3.691 | 0.331 |

Supplementary Table 10d: Multiple linear regression of Frailty Phenotype score by LMM in females. Model 3

|  | Coefficient | Lower 95% C.I. | Upper 95% C.I. | P |
| --- | --- | --- | --- | --- |
| Low Muscle Mass |  |  |  |  |
| *No* | REFERENCE | | | |
| *Yes* | 0.242 | -0.328 | 0.813 | 0.399 |
| Age | 0.004 | -0.019 | 0.026 | 0.738 |
| Ethnicity |  |  |  |  |
| *White* | REFERENCE | | | |
| *South Asian* | 0.394 | -0.554 | 1.343 | 0.409 |
| *Black* | -0.338 | -1.042 | 0.367 | 0.342 |
| *Other* | -0.359 | -2.047 | 1.330 | 0.672 |
| Education Level |  |  |  |  |
| *High School* | REFERENCE | | | |
| *College/6th form* | 0.352 | -0.256 | 0.961 | 0.251 |
| *University* | -0.149 | -1.058 | 0.760 | 0.744 |
| IMD Quintile |  |  |  |  |
| *1* | REFERENCE | | | |
| *2* | 0.236 | -0.483 | 0.954 | 0.514 |
| *3* | -0.165 | -0.996 | 0.667 | 0.694 |
| *4* | -0.915 | -1.728 | -0.102 | 0.028 |
| *5* | -1.022 | -1.817 | -0.226 | 0.013 |
| *Unknown* | 0.227 | -0.829 | 1.283 | 0.668 |
| Social Support |  |  |  |  |
| *Yes* | REFERENCE | | | |
| *No* | -0.097 | -1.192 | 0.997 | 0.859 |
| HD vintage (months) | -0.001 | -0.007 | 0.005 | 0.687 |
| Charlson Index* | -0.015 | -0.197 | 0.167 | 0.868 |
| Cognitive Impairment |  |  |  |  |
| *No* | REFERENCE | | | |
| *Yes* | 0.304 | -0.338 | 0.946 | 0.348 |
| PHQ-9 score | 0.047 | -0.009 | 0.102 | 0.098 |
| Self-reported health change |  |  |  |  |
| *Better* | REFERENCE | | | |
| *The Same* | -0.094 | -0.909 | 0.721 | 0.818 |
| *Worse* | -0.296 | -1.106 | 0.515 | 0.468 |
| Self-reported health today/100 | -0.005 | -0.019 | 0.009 | 0.507 |
| Walking Aid Use |  |  |  |  |
| *No* | REFERENCE | | | |
| *Yes* | 1.062 | 0.543 | 1.580 | <0.001 |
| Self-reported slow walking |  |  |  |  |
| *No* | REFERENCE | | | |
| *Yes* | 0.702 | 0.172 | 1.232 | 0.010 |
| Constant | 1.090 | -0.988 | 3.167 | 0.298 |

Supplementary Table 11a: Simple linear regression of frailty phenotype score by sarcopenia (EWGSOP definition) in males.

|  | Coefficient | Lower 95% C.I. | Upper 95% C.I. | P |
| --- | --- | --- | --- | --- |
| Sarcopenia |  |  |  |  |
| *No* | REFERENCE | | | |
| *Yes* | 1.456 | 1.038 | 1.874 | <0.001 |
| Constant | 1.364 | 1.112 | 1.615 | <0.001 |

Supplementary Table 11b: Multiple linear regression of frailty phenotype score by sarcopenia (EWGSOP definition) in males. Model 1

|  | Coefficient | Lower 95% C.I. | Upper 95% C.I. | P |
| --- | --- | --- | --- | --- |
| Sarcopenia |  |  |  |  |
| *No* | REFERENCE | | | |
| *Yes* | 1.387 | 0.912 | 1.863 | <0.001 |
| Age | 0.016 | -0.002 | 0.033 | 0.077 |
| Ethnicity |  |  |  |  |
| *White* | REFERENCE | | | |
| *South Asian* | 0.151 | -0.359 | 0.661 | 0.558 |
| *Black* | -0.282 | -0.937 | 0.374 | 0.397 |
| *Other* | -0.681 | -2.577 | 1.215 | 0.479 |
| Education Level | |  |  |  |
| *High School* | REFERENCE | | | |
| *College/6th form* | -0.080 | -0.611 | 0.451 | 0.766 |
| *University* | -0.478 | -1.251 | 0.294 | 0.223 |
| IMD Quintile |  |  |  |  |
| *1* | REFERENCE | | | |
| *2* | -0.493 | -1.156 | 0.170 | 0.144 |
| *3* | -0.012 | -0.606 | 0.582 | 0.968 |
| *4* | -0.063 | -0.956 | 0.830 | 0.889 |
| *5* | -0.444 | -1.218 | 0.330 | 0.258 |
| *Unknown* | -0.896 | -1.717 | -0.076 | 0.033 |
| Social Support |  |  |  |  |
| *Yes* | REFERENCE | | | |
| *No* | -0.043 | -0.916 | 0.830 | 0.923 |
| HD vintage (months) | -0.003 | -0.007 | 0.001 | 0.158 |
| Constant | 0.792 | -0.330 | 1.914 | 0.165 |

Supplementary Table 11c: Multiple linear regression of frailty phenotype score by sarcopenia (EWGSOP definition) in males. Model 2

|  | Coefficient | Lower 95% C.I. | Upper 95% C.I. | P |
| --- | --- | --- | --- | --- |
| Sarcopenia |  |  |  |  |
| *No* | REFERENCE | | | |
| *Yes* | 0.847 | 0.397 | 1.298 | <0.001 |
| Age | 0.017 | -0.003 | 0.038 | 0.101 |
| Ethnicity |  |  |  |  |
| *White* | REFERENCE | | | |
| *South Asian* | 0.382 | -0.107 | 0.871 | 0.124 |
| *Black* | -0.207 | -0.764 | 0.349 | 0.462 |
| *Other* | -1.270 | -2.887 | 0.347 | 0.122 |
| Education Level | |  |  |  |
| *High School* | REFERENCE | | | |
| *College/6th form* | -0.040 | -0.511 | 0.432 | 0.868 |
| *University* | -0.292 | -0.982 | 0.397 | 0.402 |
| IMD Quintile |  |  |  |  |
| *1* | REFERENCE | | | |
| *2* | 0.003 | -0.608 | 0.614 | 0.993 |
| *3* | 0.104 | -0.408 | 0.617 | 0.687 |
| *4* | -0.193 | -0.983 | 0.597 | 0.629 |
| *5* | -0.140 | -0.825 | 0.545 | 0.687 |
| *Unknown* | -0.302 | -1.076 | 0.471 | 0.440 |
| Social Support |  |  |  |  |
| *Yes* | REFERENCE | | | |
| *No* | -0.433 | -1.230 | 0.364 | 0.284 |
| HD vintage (months) | -0.002 | -0.005 | 0.001 | 0.252 |
| Charlson Index* | 0.059 | -0.048 | 0.165 | 0.277 |
| Cognitive Impairment | |  |  |  |
| *No* | REFERENCE | | | |
| *Yes* | -0.114 | -0.635 | 0.406 | 0.664 |
| PHQ-9 score | 0.065 | 0.023 | 0.107 | 0.002 |
| Self-reported health change | |  |  |  |
| *Better* | REFERENCE | | | |
| *The Same* | -0.135 | -0.664 | 0.395 | 0.616 |
| *Worse* | 0.285 | -0.221 | 0.792 | 0.266 |
| Self-reported health today/100 | -0.018 | -0.028 | -0.008 | <0.001 |
| Constant | 1.073 | -0.299 | 2.444 | 0.124 |

Supplementary Table 11d: Multiple linear regression of frailty phenotype score by sarcopenia (EWGSOP definition) in males. Model 3

|  | Coefficient | Lower 95% C.I. | Upper 95% C.I. | P |
| --- | --- | --- | --- | --- |
| Sarcopenia |  |  |  |  |
| *No* | REFERENCE | | | |
| *Yes* | 0.822 | 0.425 | 1.220 | <0.001 |
| Age | 0.002 | -0.017 | 0.020 | 0.869 |
| Ethnicity |  |  |  |  |
| *White* | REFERENCE | | | |
| *South Asian* | 0.389 | -0.038 | 0.815 | 0.074 |
| *Black* | -0.043 | -0.549 | 0.463 | 0.866 |
| *Other* | -1.519 | -2.936 | -0.101 | 0.036 |
| Education Level | |  |  |  |
| *High School* | REFERENCE | | | |
| *College/6th form* | -0.199 | -0.628 | 0.229 | 0.358 |
| *University* | 0.118 | -0.501 | 0.737 | 0.705 |
| IMD Quintile |  |  |  |  |
| *1* | REFERENCE | | | |
| *2* | 0.038 | -0.498 | 0.574 | 0.888 |
| *3* | 0.224 | -0.227 | 0.676 | 0.327 |
| *4* | 0.055 | -0.642 | 0.751 | 0.876 |
| *5* | 0.005 | -0.598 | 0.607 | 0.987 |
| *Unknown* | -0.094 | -0.775 | 0.587 | 0.785 |
| Social Support |  |  |  |  |
| *Yes* | REFERENCE | | | |
| *No* | -0.492 | -1.195 | 0.211 | 0.168 |
| HD vintage (months) | -0.001 | -0.004 | 0.002 | 0.706 |
| Charlson Index* | 0.058 | -0.035 | 0.151 | 0.223 |
| Cognitive Impairment | |  |  |  |
| *No* | REFERENCE | | | |
| *Yes* | -0.250 | -0.709 | 0.210 | 0.283 |
| PHQ-9 score | 0.060 | 0.023 | 0.097 | 0.002 |
| Self-reported health change | |  |  |  |
| *Better* | REFERENCE | | | |
| *The Same* | -0.191 | -0.655 | 0.274 | 0.417 |
| *Worse* | 0.091 | -0.357 | 0.538 | 0.688 |
| Self-reported health today/100 | -0.009 | -0.018 | 0.000 | 0.057 |
| Walking Aid Use | |  |  |  |
| *No* | REFERENCE | | | |
| *Yes* | 0.757 | 0.363 | 1.151 | <0.001 |
| Self-rated slow walking | |  |  |  |
| *No* | REFERENCE | | | |
| *Yes* | 0.688 | 0.279 | 1.096 | <0.001 |
| Constant | 0.893 | -0.316 | 2.102 | 0.146 |

Supplementary Table 12a: Simple linear regression of frailty phenotype score by sarcopenia (EWGSOP definition) in females.

|  | Coefficient | Lower 95% C.I. | Upper 95% C.I. | P |
| --- | --- | --- | --- | --- |
| Sarcopenia |  |  |  |  |
| *No* | REFERENCE | | | |
| *Yes* | 0.824 | 0.192 | 1.456 | 0.011 |
| Constant | 1.891 | 1.576 | 2.205 | <0.001 |

Supplementary Table 12b: Multiple linear regression of frailty phenotype score by sarcopenia (EWGSOP definition) in females. Model 1

|  | Coefficient | Lower 95% C.I. | Upper 95% C.I. | P |
| --- | --- | --- | --- | --- |
| Sarcopenia |  |  |  |  |
| *No* | REFERENCE | | | |
| *Yes* | 0.633 | -0.053 | 1.320 | 0.070 |
| Age | 0.010 | -0.010 | 0.029 | 0.323 |
| Ethnicity |  |  |  |  |
| *White* | REFERENCE | | | |
| *South Asian* | 0.233 | -0.696 | 1.161 | 0.619 |
| *Black* | -0.401 | -1.248 | 0.446 | 0.348 |
| *Other* | -1.550 | -3.505 | 0.405 | 0.118 |
| Education Level | |  |  |  |
| *High School* | REFERENCE | | | |
| *College/6th form* | 0.406 | -0.269 | 1.080 | 0.234 |
| *University* | -0.204 | -1.209 | 0.801 | 0.687 |
| IMD Quintile |  |  |  |  |
| *1* | REFERENCE | | | |
| *2* | 0.290 | -0.529 | 1.108 | 0.482 |
| *3* | -0.474 | -1.427 | 0.478 | 0.324 |
| *4* | -1.205 | -2.152 | -0.257 | 0.013 |
| *5* | -1.055 | -2.003 | -0.108 | 0.030 |
| *Unknown* | 0.605 | -0.594 | 1.804 | 0.318 |
| Social Support |  |  |  |  |
| *Yes* | REFERENCE | | | |
| *No* | -0.039 | -1.235 | 1.156 | 0.948 |
| HD vintage (months) | -0.002 | -0.008 | 0.005 | 0.627 |
| Constant | 1.632 | 0.226 | 3.039 | 0.024 |

Supplementary Table 12c: Multiple linear regression of frailty phenotype score by sarcopenia (EWGSOP definition) in females. Model 2

|  | Coefficient | Lower 95% C.I. | Upper 95% C.I. | P |
| --- | --- | --- | --- | --- |
| Sarcopenia |  |  |  |  |
| *No* | REFERENCE | | | |
| *Yes* | 0.756 | 0.038 | 1.474 | 0.039 |
| Age | 0.011 | -0.016 | 0.037 | 0.424 |
| Ethnicity |  |  |  |  |
| *White* | REFERENCE | | | |
| *South Asian* | 0.784 | -0.311 | 1.878 | 0.157 |
| *Black* | -0.148 | -0.992 | 0.697 | 0.728 |
| *Other* | -0.795 | -2.790 | 1.201 | 0.429 |
| Education Level | |  |  |  |
| *High School* | REFERENCE | | | |
| *College/6th form* | 0.548 | -0.148 | 1.245 | 0.121 |
| *University* | -0.162 | -1.233 | 0.909 | 0.764 |
| IMD Quintile |  |  |  |  |
| *1* | REFERENCE | | | |
| *2* | 0.243 | -0.602 | 1.088 | 0.567 |
| *3* | -0.341 | -1.326 | 0.644 | 0.491 |
| *4* | -0.805 | -1.789 | 0.178 | 0.107 |
| *5* | -0.803 | -1.743 | 0.138 | 0.093 |
| *Unknown* | 0.592 | -0.683 | 1.867 | 0.357 |
| Social Support |  |  |  |  |
| *Yes* | REFERENCE | | | |
| *No* | 0.116 | -1.151 | 1.383 | 0.855 |
| HD vintage (months) | -0.002 | -0.009 | 0.005 | 0.530 |
| Charlson Index* | 0.103 | -0.108 | 0.315 | 0.331 |
| Cognitive Impairment | |  |  |  |
| *No* | REFERENCE | | | |
| *Yes* | 0.291 | -0.466 | 1.048 | 0.445 |
| PHQ-9 score | 0.078 | 0.014 | 0.142 | 0.017 |
| Self-reported health change | |  |  |  |
| *Better* | REFERENCE | | | |
| *The Same* | -0.287 | -1.249 | 0.675 | 0.553 |
| *Worse* | -0.493 | -1.450 | 0.464 | 0.307 |
| Self-reported health today/100 | -0.010 | -0.026 | 0.006 | 0.219 |
| Constant | 1.099 | -1.309 | 3.508 | 0.365 |

Supplementary Table 12d: Multiple linear regression of frailty phenotype score by sarcopenia (EWGSOP definition) in females. Model 3

|  | Coefficient | Lower 95% C.I. | Upper 95% C.I. | P |
| --- | --- | --- | --- | --- |
| Sarcopenia |  |  |  |  |
| *No* | REFERENCE | | | |
| *Yes* | 0.498 | -0.108 | 1.103 | 0.105 |
| Age | 0.004 | -0.018 | 0.026 | 0.719 |
| Ethnicity |  |  |  |  |
| *White* | REFERENCE | | | |
| *South Asian* | 0.421 | -0.498 | 1.339 | 0.363 |
| *Black* | -0.259 | -0.961 | 0.443 | 0.463 |
| *Other* | -0.481 | -2.143 | 1.181 | 0.564 |
| Education Level | |  |  |  |
| *High School* | REFERENCE | | | |
| *College/6th form* | 0.353 | -0.228 | 0.934 | 0.229 |
| *University* | -0.066 | -0.953 | 0.821 | 0.883 |
| IMD Quintile |  |  |  |  |
| *1* | REFERENCE | | | |
| *2* | 0.255 | -0.446 | 0.956 | 0.469 |
| *3* | -0.116 | -0.935 | 0.704 | 0.778 |
| *4* | -0.772 | -1.589 | 0.045 | 0.064 |
| *5* | -1.034 | -1.816 | -0.251 | 0.010 |
| *Unknown* | 0.368 | -0.690 | 1.426 | 0.489 |
| Social Support |  |  |  |  |
| *Yes* | REFERENCE | | | |
| *No* | -0.147 | -1.227 | 0.932 | 0.786 |
| HD vintage (months) | -0.002 | -0.007 | 0.004 | 0.584 |
| Charlson Index* | -0.002 | -0.181 | 0.177 | 0.984 |
| Cognitive Impairment | |  |  |  |
| *No* | REFERENCE | | | |
| *Yes* | 0.331 | -0.300 | 0.963 | 0.298 |
| PHQ-9 score | 0.049 | -0.005 | 0.103 | 0.072 |
| Self-reported health change | |  |  |  |
| *Better* | REFERENCE | | | |
| *The Same* | -0.154 | -0.951 | 0.644 | 0.701 |
| *Worse* | -0.358 | -1.152 | 0.435 | 0.370 |
| Self-reported health today/100 | -0.004 | -0.017 | 0.010 | 0.581 |
| Walking Aid Use | |  |  |  |
| *No* | REFERENCE | | | |
| *Yes* | 1.005 | 0.489 | 1.522 | <0.001 |
| Self-rated slow walking | |  |  |  |
| *No* | REFERENCE | | | |
| *Yes* | 0.725 | 0.205 | 1.245 | 0.007 |
| Constant | 0.928 | -1.108 | 2.964 | 0.366 |

Supplementary Table 13a: Logistic Regression of FP frailty by BATT in males. Univariable

|  | OR | Lower 95% C.I. | Upper 95% C.I. | P |
| --- | --- | --- | --- | --- |
| BATT | 0.936 | 0.906 | 0.968 | <0.001 |
| Constant | 10.550 | 2.183 | 50.987 | 0.003 |

Supplementary Table 13b: Multivariable Logistic Regression of FP frailty by BATT in males. Model 1

|  | OR | Lower 95% C.I. | Upper 95% C.I. | P |
| --- | --- | --- | --- | --- |
| BATT | 0.933 | 0.898 | 0.969 | <0.001 |
| Age | 1.046 | 1.004 | 1.090 | 0.032 |
| Ethnicity |  |  |  |  |
| *White* | REFERENCE | | | |
| *South Asian* | 1.020 | 0.352 | 2.951 | 0.971 |
| *Black* | 0.409 | 0.089 | 1.883 | 0.251 |
| *Other* | OMITTED | | | |
| Education Level |  |  |  |  |
| *High School* | REFERENCE | | | |
| *College/6th form* | 0.586 | 0.183 | 1.869 | 0.366 |
| *University* | OMITTED | | | |
| IMD Quintile |  |  |  |  |
| *1* | REFERENCE | | | |
| *2* | 0.385 | 0.090 | 1.646 | 0.198 |
| *3* | 0.317 | 0.089 | 1.128 | 0.076 |
| *4* | 0.719 | 0.103 | 5.030 | 0.740 |
| *5* | 0.215 | 0.040 | 1.166 | 0.075 |
| *Unknown* | 0.271 | 0.033 | 2.211 | 0.223 |
| Social Support |  |  |  |  |
| *Yes* | REFERENCE | | | |
| *No* | 0.759 | 0.116 | 4.964 | 0.774 |
| HD vintage (months) | 0.999 | 0.992 | 1.006 | 0.749 |
| Constant | 1.915 | 0.062 | 58.702 | 0.710 |

Supplementary Table 13c:: Multivariable Logistic Regression of FP frailty by BATT in males. Model 2

|  | OR | Lower 95% C.I. | Upper 95% C.I. | P |
| --- | --- | --- | --- | --- |
| BATT | 0.922 | 0.876 | 0.971 | 0.002 |
| Age | 1.097 | 1.014 | 1.187 | 0.021 |
| Ethnicity |  |  |  |  |
| *White* | REFERENCE | | | |
| *South Asian* | 2.625 | 0.528 | 13.046 | 0.238 |
| *Black* | 0.514 | 0.089 | 2.978 | 0.457 |
| *Other* | OMITTED | | | |
| Education Level |  |  |  |  |
| *High School* | REFERENCE | | | |
| *College/6th form* | 0.308 | 0.073 | 1.303 | 0.109 |
| *University* | OMITTED | | | |
| IMD Quintile |  |  |  |  |
| *1* | REFERENCE | | | |
| *2* | 1.747 | 0.316 | 9.656 | 0.522 |
| *3* | 0.443 | 0.093 | 2.118 | 0.308 |
| *4* | 0.419 | 0.038 | 4.581 | 0.476 |
| *5* | 0.207 | 0.023 | 1.886 | 0.162 |
| *Unknown* | 0.151 | 0.004 | 5.327 | 0.299 |
| Social Support |  |  |  |  |
| *Yes* | REFERENCE | | | |
| *No* | 0.243 | 0.017 | 3.570 | 0.302 |
| HD vintage (months) | 0.997 | 0.988 | 1.006 | 0.524 |
| Charlson Index* | 1.020 | 0.753 | 1.380 | 0.900 |
| Cognitive Impairment |  |  |  |  |
| *No* | REFERENCE | | | |
| *Yes* | 0.440 | 0.074 | 2.610 | 0.366 |
| PHQ-9 score | 1.144 | 0.996 | 1.314 | 0.058 |
| Self-reported health change |  |  |  |  |
| *Better* | REFERENCE | | | |
| *The Same* | 0.791 | 0.144 | 4.348 | 0.788 |
| *Worse* | 1.475 | 0.312 | 6.974 | 0.624 |
| Self-reported health today/100 | 0.954 | 0.921 | 0.988 | 0.009 |
| Constant | 1.352 | 0.008 | 219.061 | 0.907 |

Supplementary Table 13d: Multivariable Logistic Regression of FP frailty by BATT in males. Model 3

|  | OR | Lower 95% C.I. | Upper 95% C.I. | P |
| --- | --- | --- | --- | --- |
| BATT | 0.935 | 0.884 | 0.988 | 0.017 |
| Age | 1.062 | 0.972 | 1.161 | 0.183 |
| Ethnicity |  |  |  |  |
| *White* | REFERENCE | | | |
| *South Asian* | 4.935 | 0.462 | 52.658 | 0.186 |
| *Black* | 0.496 | 0.050 | 4.906 | 0.549 |
| *Other* | OMITTED | | | |
| Education Level |  |  |  |  |
| *High School* | REFERENCE | | | |
| *College/6th form* | 0.128 | 0.022 | 0.761 | 0.024 |
| *University* | OMITTED | | | |
| IMD Quintile |  |  |  |  |
| *1* | REFERENCE | | | |
| *2* | 3.335 | 0.314 | 35.402 | 0.318 |
| *3* | 0.903 | 0.137 | 5.943 | 0.915 |
| *4* | 0.551 | 0.033 | 9.276 | 0.679 |
| *5* | 0.170 | 0.012 | 2.439 | 0.192 |
| *Unknown* | 0.511 | 0.001 | 209.505 | 0.827 |
| Social Support |  |  |  |  |
| *Yes* | REFERENCE | | | |
| *No* | 0.136 | 0.007 | 2.774 | 0.194 |
| HD vintage (months) | 1.005 | 0.994 | 1.017 | 0.374 |
| Charlson Index* | 1.157 | 0.789 | 1.698 | 0.455 |
| Cognitive Impairment |  |  |  |  |
| *No* | REFERENCE | | | |
| *Yes* | 0.130 | 0.011 | 1.599 | 0.111 |
| PHQ-9 score | 1.139 | 0.955 | 1.357 | 0.147 |
| Self-reported health change |  |  |  |  |
| *Better* | REFERENCE | | | |
| *The Same* | 1.876 | 0.200 | 17.598 | 0.582 |
| *Worse* | 1.121 | 0.167 | 7.502 | 0.906 |
| Self-reported health today/100 | 0.968 | 0.930 | 1.008 | 0.112 |
| Walking Aid Use |  |  |  |  |
| *No* | REFERENCE | | | |
| *Yes* | 6.594 | 1.438 | 30.238 | 0.015 |
| Self-reported slow walking |  |  |  |  |
| *No* | REFERENCE | | | |
| *Yes* | 46.923 | 2.815 | 782.145 | 0.007 |
| Constant | 0.027 | 0.000 | 14.213 | 0.258 |

Supplementary Table 14a: Logistic Regression of FP frailty by BATT in females. Univariable

|  | OR | Lower 95% C.I. | Upper 95% C.I. | P |
| --- | --- | --- | --- | --- |
| BATT | 0.976 | 0.944 | 1.009 | 0.153 |
| Constant | 1.885 | 0.404 | 8.797 | 0.420 |

Supplementary Table 14b: Multivariable Logistic Regression of FP frailty by BATT in females. Model 1

|  | OR | Lower 95% C.I. | Upper 95% C.I. | P |
| --- | --- | --- | --- | --- |
| BATT | 0.979 | 0.938 | 1.022 | 0.331 |
| Age | 1.005 | 0.966 | 1.046 | 0.803 |
| Ethnicity |  |  |  |  |
| *White* | REFERENCE | | | |
| *South Asian* | 0.991 | 0.197 | 4.978 | 0.991 |
| *Black* | 0.220 | 0.041 | 1.176 | 0.077 |
| *Other* | OMITTED | | | |
| Education Level |  |  |  |  |
| *High School* | REFERENCE | | | |
| *College/6th form* | 3.338 | 0.812 | 13.722 | 0.095 |
| *University* | 0.321 | 0.029 | 3.598 | 0.357 |
| IMD Quintile |  |  |  |  |
| *1* | REFERENCE | | | |
| *2* | 1.092 | 0.241 | 4.956 | 0.909 |
| *3* | 0.192 | 0.033 | 1.131 | 0.068 |
| *4* | 0.050 | 0.005 | 0.541 | 0.014 |
| *5* | 0.106 | 0.011 | 1.019 | 0.052 |
| *Unknown* | 2.821 | 0.324 | 24.599 | 0.348 |
| Social Support |  |  |  |  |
| *Yes* | REFERENCE | | | |
| *No* | 2.758 | 0.217 | 35.037 | 0.434 |
| HD vintage (months) | 0.999 | 0.987 | 1.011 | 0.859 |
| Constant | 2.222 | 0.063 | 78.080 | 0.660 |

Supplementary Table 14c: Multivariable Logistic Regression of FP frailty by BATT in females. Model 2

|  | OR | Lower 95% C.I. | Upper 95% C.I. | P |
| --- | --- | --- | --- | --- |
| BATT | 0.974 | 0.918 | 1.033 | 0.377 |
| Age | 0.989 | 0.923 | 1.060 | 0.756 |
| Ethnicity |  |  |  |  |
| *White* | REFERENCE | | | |
| *South Asian* | 1.038 | 0.097 | 11.133 | 0.975 |
| *Black* | 0.678 | 0.065 | 7.037 | 0.745 |
| *Other* | OMITTED | | | |
| Education Level |  |  |  |  |
| *High School* | REFERENCE | | | |
| *College/6th form* | 5.344 | 0.682 | 41.859 | 0.111 |
| *University* | 0.058 | 0.001 | 3.937 | 0.186 |
| IMD Quintile |  |  |  |  |
| *1* | REFERENCE | | | |
| *2* | 3.980 | 0.443 | 35.762 | 0.218 |
| *3* | 0.031 | 0.002 | 0.479 | 0.013 |
| *4* | 0.017 | 0.001 | 0.560 | 0.022 |
| *5* | 0.239 | 0.017 | 3.317 | 0.286 |
| *Unknown* | 4.403 | 0.242 | 79.944 | 0.316 |
| Social Support |  |  |  |  |
| *Yes* | REFERENCE | | | |
| *No* | 5.274 | 0.185 | 150.001 | 0.330 |
| HD vintage (months) | 0.995 | 0.980 | 1.010 | 0.498 |
| Charlson Index* | 1.599 | 0.851 | 3.004 | 0.145 |
| Cognitive Impairment |  |  |  |  |
| *No* | REFERENCE | | | |
| *Yes* | 0.144 | 0.011 | 1.947 | 0.145 |
| PHQ-9 score | 1.311 | 1.078 | 1.594 | 0.007 |
| Self-reported health change |  |  |  |  |
| *Better* | REFERENCE | | | |
| *The Same* | 10.396 | 0.518 | 208.461 | 0.126 |
| *Worse* | 1.078 | 0.072 | 16.073 | 0.957 |
| Self-reported health today/100 | 0.972 | 0.928 | 1.018 | 0.229 |
| Constant | 0.967 | 0.001 | 856.387 | 0.992 |

Supplementary Table 14d: Multivariable Logistic Regression of FP frailty by BATT in females. Model 3

|  | OR | Lower 95% C.I. | Upper 95% C.I. | P |
| --- | --- | --- | --- | --- |
| BATT | 1.055 | 0.934 | 1.193 | 0.388 |
| Age | 0.954 | 0.830 | 1.096 | 0.504 |
| Ethnicity |  |  |  |  |
| *White* | REFERENCE | | | |
| *South Asian* | 0.090 | 0.002 | 4.952 | 0.239 |
| *Black* | 0.001 | 0.000 | 2.055 | 0.075 |
| *Other* | OMITTED | | | |
| Education Level |  |  |  |  |
| *High School* | REFERENCE | | | |
| *College/6th form* | 3.458 | 0.063 | 189.085 | 0.543 |
| *University* | 0.018 | 0.000 | 4.833 | 0.159 |
| IMD Quintile |  |  |  |  |
| *1* | REFERENCE | | | |
| *2* | 13.726 | 0.185 | 1017.235 | 0.233 |
| *3* | 0.000 | 0.000 | 0.256 | 0.018 |
| *4* | 0.000 | 0.000 | 0.166 | 0.024 |
| *5* | 0.003 | 0.000 | 3.456 | 0.105 |
| *Unknown* | 1.920 | 0.008 | 479.054 | 0.817 |
| Social Support |  |  |  |  |
| *Yes* | REFERENCE | | | |
| *No* | 9.966 | 0.022 | 4586.582 | 0.462 |
| HD vintage (months) | 1.009 | 0.979 | 1.040 | 0.548 |
| Charlson Index* | 0.879 | 0.268 | 2.882 | 0.831 |
| Cognitive Impairment |  |  |  |  |
| *No* | REFERENCE | | | |
| *Yes* | 0.009 | 0.000 | 0.921 | 0.046 |
| PHQ-9 score | 1.406 | 0.945 | 2.093 | 0.093 |
| Self-reported health change |  |  |  |  |
| *Better* | REFERENCE | | | |
| *The Same* | 48.302 | 0.275 | 8482.714 | 0.141 |
| *Worse* | 0.155 | 0.001 | 46.694 | 0.522 |
| Self-reported health today/100 | 0.957 | 0.875 | 1.045 | 0.326 |
| Walking Aid Use |  |  |  |  |
| *No* | REFERENCE | | | |
| *Yes* | 496.100 | 2.197 | 112027.400 | 0.025 |
| Self-reported slow walking |  |  |  |  |
| *No* | REFERENCE | | | |
| *Yes* | 352.922 | 2.242 | 55555.690 | 0.023 |
| Constant | 0.217 | 0.000 | 268829.600 | 0.831 |

Supplementary Table 15a: Univariable logistic regression of FP frailty by low muscle mass in males

|  | OR | Lower 95% C.I. | Upper 95% C.I. | P |
| --- | --- | --- | --- | --- |
| Low Muscle Mass |  |  |  |  |
| *No* | REFERENCE | | | |
| *Yes* | 6.840 | 2.477 | 19 | <0.001 |
| Constant | 0.111 | 0.044 | 0.280 | <0.001 |

Supplementary Table 15b: Multivariable logistic regression of FP frailty by low muscle mass in males. Model 1

|  | OR | Lower 95% C.I. | Upper 95% C.I. | P |
| --- | --- | --- | --- | --- |
| Low Muscle Mass |  |  |  |  |
| *No* | REFERENCE | | | |
| *Yes* | 9.136 | 2.820 | 30 | <0.001 |
| Age | 1.055 | 1.012 | 1.100 | 0.012 |
| Ethnicity |  |  |  |  |
| *White* | REFERENCE | | | |
| *South Asian* | 1.051 | 0.361 | 3.064 | 0.927 |
| *Black* | 0.462 | 0.101 | 2.117 | 0.320 |
| *Other* | OMITTED | | | |
| Education Level |  |  |  |  |
| *High School* | REFERENCE | | | |
| *College/6th form* | 0.765 | 0.240 | 2.434 | 0.650 |
| *University* | OMITTED | | | |
| IMD Quintile |  |  |  |  |
| *1* | REFERENCE | | | |
| *2* | 0.332 | 0.078 | 1.408 | 0.135 |
| *3* | 0.259 | 0.071 | 0.954 | 0.042 |
| *4* | 0.614 | 0.081 | 4.671 | 0.638 |
| *5* | 0.113 | 0.020 | 0.632 | 0.013 |
| *Unknown* | 0.215 | 0.025 | 1.878 | 0.165 |
| Social Support |  |  |  |  |
| *Yes* | REFERENCE | | | |
| *No* | 0.690 | 0.097 | 4.930 | 0.712 |
| HD vintage (months) | 0.998 | 0.991 | 1.006 | 0.658 |
| Constant | 0.009 | 0.001 | 0.158 | 0.001 |

Supplementary Table 15c: Multivariable logistic regression of FP frailty by low muscle mass in males. Model 2

|  | OR | Lower 95% C.I. | Upper 95% C.I. | P |
| --- | --- | --- | --- | --- |
| Low Muscle Mass |  |  |  |  |
| *No* | REFERENCE | | | |
| *Yes* | 7.560 | 1.840 | 31 | 0.005 |
| Age | 1.099 | 1.019 | 1.186 | 0.014 |
| Ethnicity |  |  |  |  |
| *White* | REFERENCE | | | |
| *South Asian* | 2.649 | 0.580 | 12 | 0.209 |
| *Black* | 0.560 | 0.098 | 3.198 | 0.515 |
| *Other* | OMITTED | | | |
| Education Level |  |  |  |  |
| *High School* | REFERENCE | | | |
| *College/6th form* | 0.466 | 0.116 | 1.865 | 0.280 |
| *University* | OMITTED | | | |
| IMD Quintile |  |  |  |  |
| *1* | REFERENCE | | | |
| *2* | 1.232 | 0.231 | 6.579 | 0.807 |
| *3* | 0.360 | 0.070 | 1.857 | 0.222 |
| *4* | 0.421 | 0.037 | 4.825 | 0.487 |
| *5* | 0.123 | 0.013 | 1.169 | 0.068 |
| *Unknown* | 0.148 | 0.004 | 4.970 | 0.287 |
| Social Support |  |  |  |  |
| *Yes* | REFERENCE | | | |
| *No* | 0.330 | 0.024 | 4.460 | 0.404 |
| HD vintage (months) | 0.997 | 0.987 | 1.006 | 0.485 |
| Charlson Index* | 1.069 | 0.797 | 1.432 | 0.657 |
| Cognitive Impairment |  |  |  |  |
| *No* | REFERENCE | | | |
| *Yes* | 0.535 | 0.097 | 2.953 | 0.473 |
| PHQ-9 score | 1.115 | 0.974 | 1.277 | 0.113 |
| Self-reported health change |  |  |  |  |
| *Better* | REFERENCE | | | |
| *The Same* | 0.960 | 0.190 | 4.863 | 0.961 |
| *Worse* | 1.506 | 0.328 | 6.909 | 0.599 |
| Self-reported health today/100 | 0.957 | 0.925 | 0.990 | 0.011 |
| Constant | 0.004 | 0.000 | 0.287 | 0.012 |

Supplementary Table 15d: Multivariable logistic regression of FP frailty by low muscle mass in males. Model 3

|  | OR | Lower 95% C.I. | Upper 95% C.I. | P |
| --- | --- | --- | --- | --- |
| Low Muscle Mass |  |  |  |  |
| *No* | REFERENCE | | | |
| *Yes* | 7.290 | 1.294 | 41.067 | 0.024 |
| Age | 1.078 | 0.985 | 1.180 | 0.102 |
| Ethnicity |  |  |  |  |
| *White* | REFERENCE | | | |
| *South Asian* | 6.403 | 0.671 | 61 | 0.107 |
| *Black* | 0.645 | 0.068 | 6.134 | 0.703 |
| *Other* | OMITTED | | | |
| Education Level |  |  |  |  |
| *High School* | REFERENCE | | | |
| *College/6th form* | 0.215 | 0.038 | 1.208 | 0.081 |
| *University* | OMITTED | | | |
| IMD Quintile |  |  |  |  |
| *1* | REFERENCE | | | |
| *2* | 2.464 | 0.260 | 23 | 0.432 |
| *3* | 0.645 | 0.089 | 4.648 | 0.663 |
| *4* | 0.387 | 0.022 | 6.848 | 0.517 |
| *5* | 0.084 | 0.005 | 1.393 | 0.084 |
| *Unknown* | 0.322 | 0.002 | 59 | 0.670 |
| Social Support |  |  |  |  |
| *Yes* | REFERENCE | | | |
| *No* | 0.118 | 0.007 | 2.027 | 0.141 |
| HD vintage (months) | 1.005 | 0.992 | 1.019 | 0.449 |
| Charlson Index* | 1.224 | 0.844 | 1.775 | 0.287 |
| Cognitive Impairment |  |  |  |  |
| *No* | REFERENCE | | | |
| *Yes* | 0.122 | 0.011 | 1.309 | 0.082 |
| PHQ-9 score | 1.133 | 0.955 | 1.344 | 0.152 |
| Self-reported health change |  |  |  |  |
| *Better* | REFERENCE | | | |
| *The Same* | 1.772 | 0.206 | 15 | 0.602 |
| *Worse* | 0.935 | 0.150 | 5.846 | 0.943 |
| Self-reported health today/100 | 0.971 | 0.934 | 1.010 | 0.144 |
| Walking Aid Use |  |  |  |  |
| *No* | REFERENCE | | | |
| *Yes* | 5.372 | 1.197 | 24 | 0.028 |
| Self-reported slow walking |  |  |  |  |
| *No* | REFERENCE | | | |
| *Yes* | 63.511 | 4.263 | 946 | 0.003 |
| Constant | 0.000 | 0.000 | 0.040 | 0.003 |

Supplementary Table 16a: Univariable logistic regression of FP frailty by low muscle mass in females

|  | OR | Lower 95% C.I. | Upper 95% C.I. | P |
| --- | --- | --- | --- | --- |
| Low Muscle Mass |  |  |  |  |
| *No* | REFERENCE | | | |
| *Yes* | 2.884 | 1.152 | 7.217 | 0.024 |
| Constant | 0.421 | 0.235 | 0.755 | 0.004 |

Supplementary Table 16b: Multivariable logistic regression of FP frailty by low muscle mass in females. Model 1

|  | OR | Lower 95% C.I. | Upper 95% C.I. | P |
| --- | --- | --- | --- | --- |
| Low Muscle Mass |  |  |  |  |
| *No* | REFERENCE | | | |
| *Yes* | 2.888 | 0.884 | 9.432 | 0.079 |
| Age | 1.005 | 0.966 | 1.046 | 0.795 |
| Ethnicity |  |  |  |  |
| *White* | REFERENCE | | | |
| *South Asian* | 0.980 | 0.186 | 5.154 | 0.981 |
| *Black* | 0.244 | 0.046 | 1.295 | 0.098 |
| *Other* | OMITTED | | | |
| Education Level |  |  |  |  |
| *High School* | REFERENCE | | | |
| *College/6th form* | 3.513 | 0.863 | 14 | 0.079 |
| *University* | 0.384 | 0.034 | 4.300 | 0.437 |
| IMD Quintile |  |  |  |  |
| *1* | REFERENCE | | | |
| *2* | 0.950 | 0.200 | 4.511 | 0.948 |
| *3* | 0.174 | 0.028 | 1.096 | 0.063 |
| *4* | 0.047 | 0.004 | 0.505 | 0.012 |
| *5* | 0.100 | 0.010 | 0.998 | 0.050 |
| *Unknown* | 3.074 | 0.336 | 28 | 0.320 |
| Social Support |  |  |  |  |
| *Yes* | REFERENCE | | | |
| *No* | 2.484 | 0.157 | 39 | 0.519 |
| HD vintage (months) | 0.998 | 0.986 | 1.010 | 0.694 |
| Constant | 0.618 | 0.040 | 9.645 | 0.731 |

Supplementary Table 16c: Multivariable logistic regression of FP frailty by low muscle mass in females. Model 2

|  | OR | Lower 95% C.I. | Upper 95% C.I. | P |
| --- | --- | --- | --- | --- |
| Low Muscle Mass |  |  |  |  |
| *No* | REFERENCE | | | |
| *Yes* | 3.925 | 0.739 | 21 | 0.109 |
| Age | 0.990 | 0.923 | 1.061 | 0.769 |
| Ethnicity |  |  |  |  |
| *White* | REFERENCE | | | |
| *South Asian* | 1.538 | 0.132 | 18 | 0.731 |
| *Black* | 0.765 | 0.074 | 7.956 | 0.823 |
| *Other* | OMITTED | | | |
| Education Level |  |  |  |  |
| *High School* | REFERENCE | | | |
| *College/6th form* | 6.504 | 0.774 | 55 | 0.085 |
| *University* | 0.100 | 0.001 | 7.433 | 0.295 |
| IMD Quintile |  |  |  |  |
| *1* | REFERENCE | | | |
| *2* | 3.314 | 0.350 | 31 | 0.296 |
| *3* | 0.041 | 0.003 | 0.626 | 0.022 |
| *4* | 0.019 | 0.001 | 0.607 | 0.025 |
| *5* | 0.172 | 0.011 | 2.719 | 0.211 |
| *Unknown* | 5.069 | 0.240 | 107 | 0.297 |
| Social Support |  |  |  |  |
| *Yes* | REFERENCE | | | |
| *No* | 4.949 | 0.152 | 161 | 0.368 |
| HD vintage (months) | 0.992 | 0.977 | 1.008 | 0.321 |
| Charlson Index* | 1.658 | 0.872 | 3.151 | 0.123 |
| Cognitive Impairment |  |  |  |  |
| *No* | REFERENCE | | | |
| *Yes* | 0.159 | 0.011 | 2.363 | 0.182 |
| PHQ-9 score | 1.327 | 1.086 | 1.621 | 0.006 |
| Self-reported health change |  |  |  |  |
| *Better* | REFERENCE | | | |
| *The Same* | 9.225 | 0.392 | 217 | 0.168 |
| *Worse* | 1.004 | 0.061 | 17 | 0.998 |
| Self-reported health today/100 | 0.977 | 0.931 | 1.025 | 0.339 |
| Constant | 0.107 | 0.000 | 125 | 0.536 |

Supplementary Table 16d: Multivariable logistic regression of FP frailty by low muscle mass in females. Model 3

|  | OR | Lower 95% C.I. | Upper 95% C.I. | P |
| --- | --- | --- | --- | --- |
| Low Muscle Mass |  |  |  |  |
| *No* | REFERENCE | | | |
| *Yes* | 2.717 | 0.144 | 51 | 0.505 |
| Age | 0.947 | 0.823 | 1.090 | 0.449 |
| Ethnicity |  |  |  |  |
| *White* | REFERENCE | | | |
| *South Asian* | 0.104 | 0.002 | 6.464 | 0.283 |
| *Black* | 0.006 | 0.000 | 3.503 | 0.115 |
| *Other* | OMITTED | | | |
| Education Level |  |  |  |  |
| *High School* | REFERENCE | | | |
| *College/6th form* | 5.546 | 0.110 | 281 | 0.392 |
| *University* | 0.138 | 0.001 | 29 | 0.469 |
| IMD Quintile |  |  |  |  |
| *1* | REFERENCE | | | |
| *2* | 15.222 | 0.177 | 1311 | 0.231 |
| *3* | 0.002 | 0.000 | 0.299 | 0.016 |
| *4* | 0.000 | 0.000 | 0.097 | 0.014 |
| *5* | 0.007 | 0.000 | 6.298 | 0.154 |
| *Unknown* | 5.282 | 0.026 | 1093 | 0.541 |
| Social Support |  |  |  |  |
| *Yes* | REFERENCE | | | |
| *No* | 6.457 | 0.017 | 2395 | 0.537 |
| HD vintage (months) | 1.001 | 0.973 | 1.029 | 0.966 |
| Charlson Index* | 0.889 | 0.291 | 2.714 | 0.836 |
| Cognitive Impairment |  |  |  |  |
| *No* | REFERENCE | | | |
| *Yes* | 0.037 | 0.001 | 2.030 | 0.107 |
| PHQ-9 score | 1.436 | 0.994 | 2.074 | 0.054 |
| Self-reported health change |  |  |  |  |
| *Better* | REFERENCE | | | |
| *The Same* | 58.672 | 0.208 | 16512 | 0.157 |
| *Worse* | 0.213 | 0.001 | 58.114 | 0.589 |
| Self-reported health today/100 | 0.970 | 0.889 | 1.059 | 0.497 |
| Walking Aid Use |  |  |  |  |
| *No* | REFERENCE | | | |
| *Yes* | 235.806 | 2.699 | 20605 | 0.017 |
| Self-reported slow walking |  |  |  |  |
| *No* | REFERENCE | | | |
| *Yes* | 90.422 | 2.252 | 3630 | 0.017 |
| Constant | 0.599 | 0.000 | 167009 | 0.936 |

Supplementary Table 17a: Univariable logistic regression of FP frailty by sarcopenia (EWGSOP definition) in males.

|  | OR | Lower 95% C.I. | Upper 95% C.I. | P |
| --- | --- | --- | --- | --- |
| Sarcopenia |  |  |  |  |
| *No* | 1.000 |  |  |  |
| *Yes* | 7.299 | 3.276 | 16.262 | <0.001 |
| Constant | 0.189 | 0.107 | 0.335 | <0.001 |

Supplementary Table 17b: Multivariable logistic regression of FP frailty by sarcopenia (EWGSOP definition) in males. Model 1

|  | OR | Lower 95% C.I. | Upper 95% C.I. | P |
| --- | --- | --- | --- | --- |
| Sarcopenia |  |  |  |  |
| *No* | 1.000 |  |  |  |
| *Yes* | 7.673 | 2.854 | 20.627 | <0.001 |
| Age | 1.037 | 0.994 | 1.082 | 0.093 |
| Ethnicity |  |  |  |  |
| *White* | 1.000 |  |  |  |
| *South Asian* | 0.878 | 0.285 | 2.712 | 0.822 |
| *Black* | 0.636 | 0.138 | 2.922 | 0.561 |
| *Other* | OMITTED | | | |
| Education Level |  |  |  |  |
| *High School* | 1.000 |  |  |  |
| *College/6th form* | 0.576 | 0.174 | 1.901 | 0.365 |
| *University* | OMITTED | | | |
| IMD Quintile |  |  |  |  |
| *1* | 1.000 |  |  |  |
| *2* | 0.612 | 0.146 | 2.566 | 0.502 |
| *3* | 0.918 | 0.264 | 3.196 | 0.893 |
| *4* | 0.612 | 0.078 | 4.789 | 0.640 |
| *5* | 0.372 | 0.064 | 2.170 | 0.272 |
| *Unknown* | 0.238 | 0.030 | 1.910 | 0.177 |
| Social Support |  |  |  |  |
| *Yes* | 1.000 |  |  |  |
| *No* | 0.595 | 0.086 | 4.100 | 0.598 |
| HD vintage (months) | 0.998 | 0.991 | 1.006 | 0.623 |
| Constant | 0.038 | 0.002 | 0.655 | 0.024 |

Supplementary Table 17c: Multivariable logistic regression of FP frailty by sarcopenia (EWGSOP definition) in males. Model 2

|  | OR | Lower 95% C.I. | Upper 95% C.I. | P |
| --- | --- | --- | --- | --- |
| Sarcopenia |  |  |  |  |
| *No* | 1.000 |  |  |  |
| *Yes* | 5.490 | 1.663 | 18.123 | 0.005 |
| Age | 1.079 | 1.003 | 1.162 | 0.042 |
| Ethnicity |  |  |  |  |
| *White* | 1.000 |  |  |  |
| *South Asian* | 2.530 | 0.545 | 11.746 | 0.236 |
| *Black* | 0.753 | 0.125 | 4.528 | 0.757 |
| *Other* | OMITTED | | | |
| Education Level |  |  |  |  |
| *High School* | 1.000 |  |  |  |
| *College/6th form* | 0.404 | 0.103 | 1.578 | 0.192 |
| *University* | OMITTED | | | |
| IMD Quintile |  |  |  |  |
| *1* | 1.000 |  |  |  |
| *2* | 2.101 | 0.358 | 12.338 | 0.411 |
| *3* | 1.103 | 0.258 | 4.703 | 0.895 |
| *4* | 0.470 | 0.047 | 4.693 | 0.520 |
| *5* | 0.394 | 0.048 | 3.202 | 0.383 |
| *Unknown* | 0.192 | 0.007 | 5.380 | 0.332 |
| Social Support |  |  |  |  |
| *Yes* | 1.000 |  |  |  |
| *No* | 0.257 | 0.018 | 3.726 | 0.319 |
| HD vintage (months) | 0.997 | 0.988 | 1.007 | 0.585 |
| Charlson Index* | 1.013 | 0.757 | 1.355 | 0.932 |
| Cognitive Impairment |  |  |  |  |
| *No* | 1.000 |  |  |  |
| *Yes* | 0.539 | 0.096 | 3.031 | 0.483 |
| PHQ-9 score | 1.100 | 0.968 | 1.249 | 0.145 |
| Self-reported health change |  |  |  |  |
| *Better* | 1.000 |  |  |  |
| *The Same* | 0.772 | 0.146 | 4.093 | 0.761 |
| *Worse* | 1.819 | 0.407 | 8.123 | 0.433 |
| Self-reported health today/100 | 0.964 | 0.933 | 0.996 | 0.026 |
| Constant | 0.015 | 0.000 | 1.038 | 0.052 |

Supplementary Table 17d: Multivariable logistic regression of FP frailty by sarcopenia (EWGSOP definition) in males. Model 3

|  | OR | Lower 95% C.I. | Upper 95% C.I. | P |
| --- | --- | --- | --- | --- |
| Sarcopenia |  |  |  |  |
| *No* | REFERENCE | | | |
| *Yes* | 9.855 | 1.800 | 53.950 | 0.008 |
| Age | 1.039 | 0.947 | 1.140 | 0.420 |
| Ethnicity |  |  |  |  |
| *White* | REFERENCE | | | |
| *South Asian* | 5.015 | 0.476 | 52.849 | 0.180 |
| *Black* | 1.277 | 0.124 | 13.143 | 0.837 |
| *Other* | OMITTED | | | |
| Education Level |  |  |  |  |
| *High School* | REFERENCE | | | |
| *College/6th form* | 0.152 | 0.025 | 0.909 | 0.039 |
| *University* | OMITTED | | | |
| IMD Quintile |  |  |  |  |
| *1* | REFERENCE | | | |
| *2* | 4.460 | 0.462 | 43.015 | 0.196 |
| *3* | 2.605 | 0.378 | 17.940 | 0.331 |
| *4* | 0.473 | 0.025 | 9.010 | 0.618 |
| *5* | 0.329 | 0.021 | 5.051 | 0.425 |
| *Unknown* | 0.421 | 0.001 | 159.369 | 0.775 |
| Social Support |  |  |  |  |
| *Yes* | REFERENCE | | | |
| *No* | 0.128 | 0.008 | 2.110 | 0.151 |
| HD vintage (months) | 1.005 | 0.993 | 1.018 | 0.395 |
| Charlson Index* | 1.221 | 0.823 | 1.811 | 0.320 |
| Cognitive Impairment |  |  |  |  |
| *No* | REFERENCE | | | |
| *Yes* | 0.160 | 0.011 | 2.407 | 0.185 |
| PHQ-9 score | 1.132 | 0.961 | 1.334 | 0.137 |
| Self-reported health change |  |  |  |  |
| *Better* | REFERENCE | | | |
| *The Same* | 1.524 | 0.176 | 13.168 | 0.702 |
| *Worse* | 1.207 | 0.191 | 7.612 | 0.841 |
| Self-reported health today/100 | 0.982 | 0.940 | 1.026 | 0.421 |
| Walking Aid Use |  |  |  |  |
| *No* | REFERENCE | | | |
| *Yes* | 10.050 | 1.852 | 54.524 | 0.007 |
| Self-rated slow walking |  |  |  |  |
| *No* | REFERENCE | | | |
| *Yes* | 71.284 | 3.876 | 1310.861 | 0.004 |
| Constant | 0.000 | 0.000 | 0.100 | 0.007 |

Supplementary Table 18a: Univariable logistic regression of FP frailty by sarcopenia (EWGSOP definition) in females.

|  | OR | Lower 95% C.I. | Upper 95% C.I. | P |
| --- | --- | --- | --- | --- |
| Sarcopenia |  |  |  |  |
| *No* | REFERENCE | | | |
| *Yes* | 4.737 | 1.651 | 13.589 | 0.004 |
| Constant | 0.422 | 0.247 | 0.722 | 0.002 |

Supplementary Table 18b: Multivariable logistic regression of FP frailty by sarcopenia (EWGSOP definition) in females. Model 1

|  | OR | Lower 95% C.I. | Upper 95% C.I. | P |
| --- | --- | --- | --- | --- |
| Sarcopenia |  |  |  |  |
| *No* | REFERENCE | | | |
| *Yes* | 4.823 | 1.225 | 18.994 | 0.024 |
| Age | 1.010 | 0.971 | 1.051 | 0.621 |
| Ethnicity |  |  |  |  |
| *White* | REFERENCE | | | |
| *South Asian* | 1.056 | 0.191 | 5.834 | 0.950 |
| *Black* | 0.283 | 0.052 | 1.551 | 0.146 |
| *Other* | OMITTED | | | |
| Education Level |  |  |  |  |
| *High School* | REFERENCE | | | |
| *College/6th form* | 3.536 | 0.855 | 14.628 | 0.081 |
| *University* | 0.424 | 0.037 | 4.818 | 0.489 |
| IMD Quintile |  |  |  |  |
| *1* | REFERENCE | | | |
| *2* | 1.044 | 0.215 | 5.075 | 0.958 |
| *3* | 0.221 | 0.035 | 1.396 | 0.108 |
| *4* | 0.067 | 0.006 | 0.712 | 0.025 |
| *5* | 0.084 | 0.008 | 0.898 | 0.040 |
| *Unknown* | 4.041 | 0.425 | 38.395 | 0.224 |
| Social Support |  |  |  |  |
| *Yes* | REFERENCE | | | |
| *No* | 1.830 | 0.093 | 35.988 | 0.691 |
| HD vintage (months) | 0.997 | 0.985 | 1.009 | 0.635 |
| Constant | 0.427 | 0.025 | 7.161 | 0.554 |

Supplementary Table 18c: Multivariable logistic regression of FP frailty by sarcopenia (EWGSOP definition) in females. Model 2

|  | OR | Lower 95% C.I. | Upper 95% C.I. | P |
| --- | --- | --- | --- | --- |
| Sarcopenia |  |  |  |  |
| *No* | REFERENCE | | | |
| *Yes* | 5.709 | 0.762 | 42.804 | 0.090 |
| Age | 0.988 | 0.920 | 1.061 | 0.735 |
| Ethnicity |  |  |  |  |
| *White* | REFERENCE | | | |
| *South Asian* | 1.261 | 0.113 | 14.047 | 0.851 |
| *Black* | 0.846 | 0.073 | 9.801 | 0.894 |
| *Other* | OMITTED | | | |
| Education Level |  |  |  |  |
| *High School* | REFERENCE | | | |
| *College/6th form* | 5.187 | 0.677 | 39.733 | 0.113 |
| *University* | 0.096 | 0.001 | 6.525 | 0.276 |
| IMD Quintile |  |  |  |  |
| *1* | REFERENCE | | | |
| *2* | 3.537 | 0.402 | 31.103 | 0.255 |
| *3* | 0.054 | 0.003 | 0.876 | 0.040 |
| *4* | 0.026 | 0.001 | 0.963 | 0.048 |
| *5* | 0.110 | 0.005 | 2.262 | 0.152 |
| *Unknown* | 8.708 | 0.359 | 211.062 | 0.183 |
| Social Support |  |  |  |  |
| *Yes* | REFERENCE | | | |
| *No* | 3.367 | 0.100 | 113.068 | 0.498 |
| HD vintage (months) | 0.991 | 0.975 | 1.007 | 0.277 |
| Charlson Index* | 1.735 | 0.896 | 3.363 | 0.102 |
| Cognitive Impairment |  |  |  |  |
| *No* | REFERENCE | | | |
| *Yes* | 0.162 | 0.011 | 2.428 | 0.188 |
| PHQ-9 score | 1.300 | 1.078 | 1.567 | 0.006 |
| Self-reported health change |  |  |  |  |
| *Better* | REFERENCE | | | |
| *The Same* | 9.149 | 0.406 | 206.277 | 0.164 |
| *Worse* | 0.968 | 0.061 | 15.371 | 0.982 |
| Self-reported health today/100 | 0.976 | 0.930 | 1.025 | 0.328 |
| Constant | 0.145 | 0.000 | 148.212 | 0.585 |

Supplementary Table 18d: Multivariable logistic regression of FP frailty by sarcopenia (EWGSOP definition) in females. Model 3

|  | OR | Lower 95% C.I. | Upper 95% C.I. | P |
| --- | --- | --- | --- | --- |
| Sarcopenia |  |  |  |  |
| *No* | REFERENCE | | | |
| *Yes* | 5.159 | 0.215 | 123.994 | 0.312 |
| Age | 0.952 | 0.830 | 1.094 | 0.490 |
| Ethnicity |  |  |  |  |
| *White* | REFERENCE | | | |
| *South Asian* | 0.081 | 0.001 | 4.956 | 0.231 |
| *Black* | 0.006 | 0.000 | 2.648 | 0.100 |
| *Other* | OMITTED | | | |
| Education Level |  |  |  |  |
| *High School* | REFERENCE | | | |
| *College/6th form* | 5.775 | 0.113 | 293.965 | 0.382 |
| *University* | 0.193 | 0.001 | 42.934 | 0.551 |
| IMD Quintile |  |  |  |  |
| *1* | REFERENCE | | | |
| *2* | 11.474 | 0.152 | 868.188 | 0.269 |
| *3* | 0.002 | 0.000 | 0.266 | 0.013 |
| *4* | 0.000 | 0.000 | 0.181 | 0.019 |
| *5* | 0.006 | 0.000 | 4.809 | 0.132 |
| *Unknown* | 6.327 | 0.033 | 1202.284 | 0.491 |
| Social Support |  |  |  |  |
| *Yes* | REFERENCE | | | |
| *No* | 5.228 | 0.014 | 2021.490 | 0.586 |
| HD vintage (months) | 0.999 | 0.973 | 1.027 | 0.965 |
| Charlson Index* | 0.845 | 0.275 | 2.595 | 0.769 |
| Cognitive Impairment |  |  |  |  |
| *No* | REFERENCE | | | |
| *Yes* | 0.051 | 0.001 | 2.552 | 0.136 |
| PHQ-9 score | 1.420 | 1.000 | 2.016 | 0.050 |
| Self-reported health change |  |  |  |  |
| *Better* | REFERENCE | | | |
| *The Same* | 43.373 | 0.203 | 9244.579 | 0.168 |
| *Worse* | 0.189 | 0.001 | 40.533 | 0.543 |
| Self-reported health today/100 | 0.974 | 0.894 | 1.060 | 0.541 |
| Walking Aid Use |  |  |  |  |
| *No* | REFERENCE | | | |
| *Yes* | 189.217 | 2.662 | 13449.290 | 0.016 |
| Self-rated slow walking |  |  |  |  |
| *No* | REFERENCE | | | |
| *Yes* | 101.312 | 2.517 | 4077.688 | 0.014 |
| Constant | 0.426 | 0.000 | 87320.960 | 0.891 |
